# Toxicological Assessment of D‐Allulose From a Novel One‐Step Fermentation Process Using Genetically Modified *Escherichia coli*: A 90‐Day Dietary Toxicity Study in Rats

**DOI:** 10.1155/jt/6597561

**Published:** 2026-03-03

**Authors:** Zinan Li, Yanmin Nie, Hongju Du, Wenjing Zhang, Shan Zheng, Wenshu Cong, Ying Feng, Guojun Li, Haiming Jing, Junyu Ning, Shan Gao

**Affiliations:** ^1^ Department of Toxicology, Beijing Center for Disease Prevention and Control, Beijing, China, chinacdc.cn; ^2^ Beijing Key Laboratory of Diagnostic and Traceability Technologies for Food Poisoning, Beijing, China; ^3^ School of Public Health, Capital Medical University, Beijing, China, ccmu.edu.cn

## Abstract

D‐Allulose is a rare monosaccharide structurally similar to D‐fructose, characterized by low caloric content and relatively high sweetness. This study aimed to conduct a 90‐day oral toxicity test to systemically evaluate the potential toxicological effects of D‐allulose produced via a novel one‐step fermentation process using genetically engineered *Escherichia coli* AS10 strain, which was genetically modified to express enzymes involved in the biosynthesis of D‐allulose from D‐glucose. The objectives were to determine its NOAEL, provide scientific evidence for the safety evaluation of this innovative food ingredient, and meet the regulatory requirements for the safety assessment of a novel process‐derived food product. A total of 96 SD rats were randomly divided into four groups (24 rats per group, with equal numbers of males and females): a control group (basal feed) and three treatment groups fed with feed containing 2.5%, 5.0%, and 10.0% D‐allulose (corresponding to estimated dietary intakes of 2000, 4000, and 8000 mg/kg body weight per day, respectively, based on an assumed daily feed intake of 8% of body weight) for 90 consecutive days. A comprehensive battery of toxicological assessments was performed on each animal, including measurements on body weight, food consumption, feed efficiency ratios, hematological metrics, serum biochemical profiles, organ weights, and histopathological examinations. No treatment‐related mortality or overt toxic symptoms were observed in any group during the study period. Statistically significant differences were noted in partial metrics (e.g., body weight, feed consumption, HCT%, PLT, ALP, TC, and absolute/relative weight of kidney) between treatment groups and control group, but these changes were deemed nontoxicologically significant due to consistency with normal physiological variability (within the historical in‐house reference ranges) or lack of corresponding pathological lesions. The NOAEL of D‐allulose was established at 8000 mg/kg BW/day in rats, confirming its safety for use as a food ingredient.

## 1. Introduction

D‐Allulose, also referred to as D‐psicose, is a rare monosaccharide found naturally in trace amounts in various botanical and primary agricultural sources. D‐Allulose is structurally similar to D‐fructose, with the key difference being the C3 position, which contains an epimeric hydroxyl group [1]. Accordingly, D‐allulose and D‐fructose share a partial absorption pathway via GLUT5 [2], while their overall absorption profiles and metabolic pathways exhibit notable differences in the human digestive and circulatory systems. Due to the absence of specific metabolic enzymes, most ingested D‐allulose is excreted in urine within 24 h, leading to minimal energy conversion [3]. D‐Allulose has a substantially lower caloric density, with an approximate caloric value of 0.8 kJ/g, compared to 17 kJ/g for sucrose [4]. Despite its reduced caloric profile, D‐allulose is almost 70% sweeter than sucrose. These properties distinguish D‐allulose as a promising alternative to regular sugars, particularly in obesity management and diabetes care applications. The low caloric content and relatively high sweetness of D‐allulose imply that it may be beneficial for people looking to reduce their caloric consumption while keeping appealing sweetness in their diets.

D‐Allulose has physicochemical qualities similar to sucrose, including solubility, texture, and heat stability [5]. As a reducing sugar, D‐allulose can participate in the Maillard reaction with amino acids, peptides, and proteins, making bakery products more visually appealing than other sugar alternatives such as erythritol [6, 7]. Several studies have highlighted the multifaceted physiological benefits of D‐allulose, including its anticariogenic potential [8], metabolic regulatory [9–13], antioxidant [14, 15], anti‐apoptotic [16], and neuroprotective properties [17]. As a novel functional monosaccharide, D‐allulose has captured considerable attention in food science and the healthcare field.

D‐Allulose was first found in wheat in the 1940s [18]. It occurs in small amounts in some fruits and processed foods such as figs, kiwifruits, dried grapes, maple syrup, condiments, and fermented sauces [19]. However, these natural sources are insufficient to meet market demand. Currently, enzyme‐catalyzed conversion from D‐fructose is the primary method for D‐allulose production [20]. The conversion of D‐fructose into D‐allulose is achieved through the utilization of specialized enzymes, such as D‐allulose‐3‐epimerase (DAE) and D‐tagatose‐3‐epimerase (DTE). These enzymes possess high substrate specificity, enabling them to selectively recognize D‐fructose molecules and catalyze the epimerization reaction at the C‐3 position, thereby facilitating the formation of D‐allulose. The enzymatic conversion process is characterized by mild reaction conditions, high regioselectivity, minimal by‐product formation, and the production of D‐allulose with high optical purity. However, the practical application of this method is challenged by several limitations, including the high cost of enzyme production, relatively low stability and susceptibility of enzymatic activity to various environmental factors, such as temperature, pH, and substrate concentration [21]. In response to these challenges, an increasing body of research has shifted its focus toward harnessing the endogenous enzymatic machinery within microbial cells for D‐allulose synthesis [22, 23]. This approach involves the genetic engineering of micro‐organisms to enhance the overexpression of enzymes involved in D‐allulose biosynthesis pathways. Subsequently, the genetically modified (GM) microbial strains are employed in fermentation processes, where specific carbohydrate substrates are metabolized by the intracellular enzymes to produce D‐allulose, which is then secreted into the extracellular medium. The “precision fermentation” strategy offers several advantages, including access to a diverse range of raw materials, a more sustainable and environmentally friendly production process and the potential for large‐scale industrial production. Nevertheless, this method presents its own set of technical challenges. The fermentation process necessitates meticulous control and optimization of multiple parameters to maximize the D‐allulose yield and conversion efficiency. Moreover, the composition of the harvested fermentation broth is inherently complex, necessitating the development and application of sophisticated separation and purification methodologies to procure high‐purity D‐allulose products [24–27]. More critically, such complexity may engender the persistence of potential biotoxins, noxious metabolites, recombinant proteins, nucleic acid fragments, cellular debris, and contaminant micro‐organisms in the final products, posing non‐negligible risks to product safety and consumer health [28]. These process‐specific risks associated with precision microbial fermentation directly necessitate targeted regulatory scrutiny and systematic safety assessment frameworks tailored to novel production methods.

In the absence of process‐specific impurities, pure D‐allulose is generally safe with low toxicity, as reviewed by Food Standards Australia New Zealand (FSANZ), with toxicological studies indicating no genotoxicity or significant reproductive/developmental harm, only manageable gastrointestinal effects at high doses, alongside established no‐observed‐adverse‐effect‐levels (NOAELs) in animal models and defined tolerable intake levels in humans [18]. Regulatory approvals for D‐allulose have been progressively granted globally. Since 2011, multiple entities have sought U.S. Food and Drug Administration (FDA) approval to classify D‐allulose as a “generally recognized as safe (GRAS)” substance, with seven of eleven applications approved to date. In August 2024, FSANZ approved D‐allulose as a novel food for specific uses. Meanwhile, countries like Japan and South Korea have also officially authorized it as a food ingredient or supplement. Most recently, the National Health Commission (NHC) of China officially approved D‐allulose as a novel food ingredient in July 2025 [29]. By contrast, the European Food Safety Authority (EFSA) has not yet confirmed its safety or issued an approval due to pending supplementary toxicological data.

However, novel production processes have the potential to induce alterations in the chemical composition, molecular structure, and nutritional content of food materials or their residues. These changes can subsequently trigger modifications in the absorption, metabolism, and toxicity profiles of these substances within the human body. In light of these inherent risks, food safety regulations in most countries have instituted mandatory safety assessment protocols for food substances derived from new or modified manufacturing processes.

Notably, although the intrinsic toxicity of pure D‐allulose as a chemical entity is determined by its molecular structure and independent of synthesis methods, the novel D‐allulose produced by GM *E. coli* via one‐step fermentation may carry “process‐specific impurities” that are not present in products from other methods. These impurities mainly include fermentation‐specific by‐products (e.g., homologous carbohydrates, residual microbial metabolites, cellular debris, and endotoxins from Gram‐negative *E. coli*), GM‐specific contaminants (e.g., residual recombinant proteins and nucleic acid fragments), and purification‐dependent impurities [25, 30, 31]. Such unique impurities could potentially alter the overall toxicological profile of the final product. Consequently, to comprehensively assess the potential toxicological effects of D‐allulose—along with its residual impurities—produced via the novel one‐step fermentation process using GM *E. coli* AS10 (CGMCC 27687), a 90‐day oral toxicity study was conducted in this research.

## 2. Materials and Methods

### 2.1. Rationale

The present study was executed in accordance with the 90‐day oral toxicity testing guideline [32], which is one of a series of technical guidelines for toxicological evaluation in food safety assessment according to National Food Safety Standards in China, and this testing guideline is also compatible with OECD Guidelines for the Testing of Chemicals Test No. 408: Repeated Dose 90‐Day Oral Toxicity Study in Rodents. Three dosing groups and one negative control group were applied in this study. Literature review and preliminary acute toxicity studies revealed that the administration of D‐allulose to experimental animals via gavage was frequently associated with adverse gastrointestinal effects, such as diarrhea and abdominal bloating [33–36]. These effects were hypothesized to be related to the hyperosmotic environment created by the D‐allulose solution within the gastrointestinal tract. To mitigate these local hyperosmotic effects and reduce gastrointestinal reactions in animals, D‐allulose was incorporated into the feed at concentrations with a maximum allowable limit. Consequently, this experiment adopted a 10% incorporation rate for the highest dosing group, with the mid‐ and low‐dose groups receiving 5% and 2.5% incorporations, respectively. Based on the assumption that a daily feed consumption is equal to 8% of body weight [32], the estimated daily intake of D‐allulose for animals in the low‐, mid‐ and high‐dose groups was calculated to be 2000, 4000, and 8000 mg/kg of body weight (BW) per day, respectively, which is as 2.2, 4.4, 8.9 times as the maximum tolerated repeated dose (0.9 g/kg bw per day) in humans according to the report from FSANZ [18].

### 2.2. Animals

In this study, a total of 96 specific pathogen‐free (SPF) Sprague‐Dawley (SD) rats, with body weights ranging from 60 to 80 g (aged 3–5 weeks) and an equal distribution of sexes, were utilized. The experimental animals were procured from SPF (Beijing) Biotechnology Co., Ltd.

The experiments were conducted within the barrier facilities designated for experimental animals at the Beijing Center for Disease Prevention and Control. The animals were housed individually in separate cages. The environmental conditions were strictly regulated, with temperatures ranging from 20°C to 26°C and relative humidity levels between 30% and 70%. A 12‐h light/dark cycle was implemented to simulate the natural day–night shift. Air cleanliness, ammonia concentration, and noise levels were monitored to guarantee compliance with the standards outlined in corresponding technical guidelines in China—Laboratory animal environment and housing facilities (GB14925).

Before the commencement of the experiment, the animals were acclimatized for 7 days in the animal housing rooms. At the end, the animals were weighed, stratified, and randomly assigned to four groups based on body weight, each consisting of 24 rats with an equal number of males and females. Throughout the experiment, the rats were housed individually in cages, with free access to a D‐allulose‐incorporated (or control) diet and water. Environmental enrichment measures such as toys were applied to minimize the stress of animals.

To ensure the welfare of experimental animals, routine care activities such as feeding, watering, and cage cleaning on a fixed schedule were conducted, which helps regulate the rats’ biological clock and reduce stress. Daily observations of the rats’ behavior, appetite, and feces were conducted, along with regular physical examinations once a week, including weight measurement and general health checks. Additionally, to decrease the rats’ fear of unfamiliar personnel, a consistent team of experimenters was maintained. The final operation protocols of animals were subjected to rigorous ethical review and granted approval by the Experimental Animal Welfare Ethics Committee of the Beijing Center for Disease Prevention and Control (Approval Number: BJCDCDL11‐2401‐7) in accordance with locally as well as internationally recognized guidelines or standards, e.g., Guidelines for Ethical Review of Animal Welfare in Experimental Research (GB/T 35892‐2018) and NIH (National Research Council) Guide for the Care and Use of Laboratory Animals.

### 2.3. Samples and Diet

D‐Allulose sample was manufactured by MicroCyto (Beijing) Biotechnology Co., Ltd. using the novel processes mentioned in the Introduction section (fermentation with *E. coli* AS10), with a purity of over 99.9%. SPF (Beijing) Biotechnology Co., Ltd., conducted the incorporation of D‐allulose‐containing feed. The feed formulation was based on a commercial diet designed for the maintenance of both adult and juvenile rodents. The D‐allulose‐containing feed premix was subjected to a series of processes, including premixing, mixing, pelleting, and drying to yield pelleted feed. Given the low caloric density of D‐allulose, the feed for each dosing group was supplemented with casein and corn oil to adjust the levels of crude protein and crude fat, the two primary nutritional components, to match those of the control group. The negative control group and the low‐, mid‐, and high‐dose groups were administered feed containing 0%, 2.5%, 5%, and 10% (w/w) D‐allulose, respectively.

### 2.4. General Clinical Observations

The clinical observation period spanned 90 days (almost 13 w), aligning with the duration of D‐allulose containing feed administration. General clinical observation was performed once a day, with meticulous documentation of any toxicological signs exhibited by the animals, including the intensity and temporality of symptoms, as well as any instances of mortality.

### 2.5. Ophthalmological Examination

Ophthalmic examination was conducted referring to the acute eye irritation test guidelines in Safety and Technical Standards for Cosmetics (2015 Edition), focusing on the cornea, conjunctiva, and iris. The examination was performed by a laboratory animal veterinarian; the conjunctiva and iris were observed for morphological abnormalities (hyperemia, edema, adhesion) and functional loss (loss of pupillary light reflex) under a 10 × magnifying glass. For corneal integrity assessment, one drop of sterile 1% fluorescein sodium solution was instilled into the lower conjunctival sac, followed by normal saline rinsing after 30 s; the absence of green fluorescence staining under magnifying glass indicated intact corneal epithelium, and the results were recorded as qualitative indicators for systemic toxicity screening. The examination was completed once before the end of the subchronic exposure period to avoid repeated operation‐induced stress.

### 2.6. Body Weight and Food Intake

Body weight and food consumption were recorded weekly to calculate the food efficiency ratio. At the end of the experiment, the body weight gain, total food intake, overall food efficiency ratio, and the actual exposure level of D‐allulose were calculated.

The formula for calculating the food efficiency ratio is as follows:

(1) food efficiency ratio=weight gainfood intake.

The formula for calculating the actual intake level of the test substance is as follows:

(2) exposure level=total food intake×incorporation concentrationday count×average body weight.

### 2.7. Animal Fasting and Urine Examination

At the end of the experiment, animals from each group were placed individually in metabolic cages for a duration of 12–14 h, during which animals were fasted with free access to water. Urine samples were collected in 50‐mL centrifuge tubes. On the subsequent day, urinalysis was conducted using the Bayer CLINITEK‐500 urinalysis meter with its commercially available, manufacturer‐matched Siemens Multistix 10 SG Reagent Strips (Siemens Healthcare Diagnostics Inc., USA). The examination parameters included appearance, specific gravity, pH, glucose, ketones, occult blood, protein, urobilinogen, bilirubin, leukocytes, and nitrites.

### 2.8. Anesthesia, Euthanasia, and Hematological Sampling

After fasting, animals were weighed and anesthetized via the intraperitoneal injection of sodium pentobarbital at a dosage of 50 mg/kg BW/day. Once the animals had attained a profound state of anesthesia, a midline laparotomy was performed to collect blood samples, both anticoagulated (using ethylene diamine tetra‐acetic acid [EDTA] or sodium citrate) and procoagulated, from the abdominal aorta. Subsequently, euthanasia was carried out by exsanguination, while the blood samples were gently inverted and mixed thoroughly.

### 2.9. Hematology and Clinical Biochemistry Assessment

Hematological and coagulation metrics were determined using a Sysmex XT‐2000i automated hematology analyzer and a Sysmex CA‐1500 automated coagulation analyzer (Sysmex Corporation, Japan), with all analyses performed using the manufacturer‐matched reagent kits (Sysmex Corporation, Japan; Siemens Healthineers Ltd.) for each analyzer in strict accordance with the standard operating instructions (SOIs) provided by the manufacturer. The detected metrics included complete blood cell counts and routine coagulation indices. The EDTA anticoagulated blood samples were specifically utilized for the quantification and classification of leukocytes, which included mononuclear cells, lymphocytes, and granulocytes, as well as the assessment of erythrocyte counts, hemoglobin (HGB) levels, hematocrit (HCT) values, and platelet (PLT) counts. The sodium citrate anticoagulated samples were dedicated to the measurement of prothrombin time (PT) and activated partial thromboplastin time (APTT), which are essential indicators of coagulation status.

The procoagulated blood samples were allowed to settle at ambient temperature for 30 min prior to centrifugation at 1200 × g for 10 min. The harvested serum was subjected to a comprehensive panel of clinical biochemical assays using a Hitachi 7600 automated biochemistry analyzer (Hitachi High‐Technologies Corporation, Japan) with the instrument‐matched, clinically registered, and certified in vitro diagnostic (IVD) reagent kits (Tellgen Corporation, China; Zhong Sheng Bei Kong Biotechnology Co., Ltd., China; Kehua Bio‐Engineering Co., Ltd., China). The measurements included alanine aminotransferase (ALT), aspartate aminotransferase (AST), alkaline phosphatase (ALP), gamma‐glutamyl transferase (GGT), urea, creatinine (Cr), glucose (Glu), total protein (TP), albumin (Alb), total cholesterol (TC), triglycerides (TG), as well as electrolytes such as chloride (Cl^−^), potassium (K^+^), and sodium (Na^+^).

### 2.10. Gross Necropsy Examination

Upon euthanasia, a prompt and thorough gross necropsy was conducted to examine the cadaver and collect pathological specimens. This examination meticulously surveyed the integumentary surface, cranium, thoracic cavity, abdominal cavity, and their constituent viscera. Attention was given to the organs’ topographical orientation, contour, dimensions, mass, coloration, adventitial coverings, consistency and the presence of exudates, hemorrhages, neoplasms, ulcerations, and any aberrant alterations. The gross pathological examination and procurement of specimens during necropsy involved a standardized panel of organs and tissues including the brain, pituitary gland, thyroid gland, thymus, lung, myocardium, liver, spleen, kidneys, adrenal glands, stomach and pancreas, duodenal, jejunal, and ileal segments, colonic and rectal segments, mesenteric lymph nodes, ovaries, uterus, testes, epididymis, prostate, urinary bladder, spinal cord (cervical, thoracic and lumbar segments), esophagus, salivary glands, cervical lymph nodes, trachea, aorta, seminal vesicles and coagulating glands, vagina, mammary tissue, femur, sternum and bone marrow, sciatic nerve, muscular tissues, skin and ocular globes, as well as any tumors or dysplastic growths. The brain, heart, thymus, adrenals, liver, kidneys, spleen, testes, epididymis, uterus, and ovaries were dissected carefully to remove connective tissues, their absolute weights were measured, and the relative organ weights were calculated (organ‐to‐body weight ratio and organ‐to‐brain weight ratio). Postcollection (or following weight measurement), the specimens were immediately submerged into 500–600 mL of a 4% formaldehyde solution for fixation and then stored in a light‐protected environment at ambient temperature for the subsequent histopathological analysis.

### 2.11. Histopathological Examination

Tissue specimens were prepared as paraffin‐embedded sections using standard histotechnique protocols, after which hematoxylin–eosin (H–E) staining was performed for microscopic histopathological assessment. Briefly, tissues were fixed in 10% neutral buffered formalin, dehydrated through a graded ethanol series, cleared in xylene, and embedded in paraffin. Serial 4‐μm sections were cut using a microtome, mounted on glass slides, and stained with H–E for microscopic histopathological evaluation. Histopathological examination was initially focused on the organs and tissues harvested from the high‐dose and vehicle control groups. When relevant toxicological indicators (e.g., clinical signs, hematological/biochemical abnormalities, or gross pathological changes) were observed, supporting histopathological examinations were conducted on the corresponding organs/tissues from the mid‐ and low‐dose groups to further clarify dose–response relationships.

### 2.12. Data Analysis and Interpretation

The statistical analysis was performed utilizing the SPSS software suite (Version 19.0). Quantitative data, such as animal body weight, food consumption, feed efficiency, hematological indices, serum biochemical profiles, organ weights, organ‐to‐body weight ratios, and organ‐to‐brain weight ratios, were described in terms of the mean ± standard deviation, and the number of subjects examined. For the data of weekly body weight, feed consumption, and food efficiency, a repeated‐measures analysis of variance (ANOVA) was performed. If the data did not meet the assumption of sphericity, a multivariate test (Pillai’s trace) was required. If there was a statistically significant difference in the group effect (dosage of the test substance), further pairwise comparisons were conducted using the least significant difference (LSD) method.

The other data except urine examination were analyzed with one‐way ANOVA, in which data not meeting the assumption of homogeneity of variance were corrected using Welch’s test; if statistical differences were found, further pairwise comparisons were conducted using the Bonferroni method (for homogeneity of variance) or the Games–Howell method (for heterogeneity of variance).

Categorical or ordinal data derived from urinalysis were detailed in frequency distributions and subjected to the Kruskal–Wallis rank sum test.

## 3. Results

### 3.1. General Clinical Observations

Throughout the observation period, no mortality was observed across all groups, and no clinical symptoms of D‐allulose toxicity were detected in animals.

In the control group, one female animal developed persistent circling behavior starting from Week 3, which was accompanied by slow weight gain. Following assessment by the laboratory animal veterinarian, the animal was diagnosed with infection‐induced otitis interna and subsequently excluded from the experiment.

### 3.2. Body Weight, Feed Consumption, Food Efficiency, and Dose Intake of D‐Allulose

The weekly body weights of the animals in each group are presented in Table 1. The ANOVA results indicated that the dosage and duration of feeding significantly influenced the body weight of both female and male animals (*p* < 0.05). Furthermore, pairwise comparisons of each level of the between‐group factor (i.e., each dosage group) revealed that the female animals in the low‐dose group (*p* < 0.05) and male animals in the mid (*p* < 0.01)‐ and high‐dose groups (*p* < 0.001) had significantly different body weight trends than their respective control groups. These findings suggest that different dosages of D‐allulose exert time‐dependent differential associations with the body weight of female and male rats. Table 2 shows the overall BW gain percentage for each animal group at the end of the experiment. The BW gain percentage of animals in each dosage group was largely consistent with the trends observed in their weekly body weights. Notably, the reduction in the weight gain rate for male animals in the mid (*p* < 0.05)‐ and high‐dose groups (*p* < 0.01) was statistically significant compared with control group.

**TABLE 1:** Body weight (g).

Gender	Group	*n*	Weeks													
			0	1	2	3	4	5	6	7	8	9	10	11	12	13
Female	Neg	11	121.9 ± 10.1	164.9 ± 10.9	195.0 ± 15.9	223.6 ± 21.8	244.8 ± 21.6	262.5 ± 25.4	278.3 ± 26.6	293.1 ± 31.1	303.7 ± 32.6	308.0 ± 31.6	318.1 ± 32.9	325.5 ± 35.6	334.1 ± 35.4	337.5 ± 34.4
	Low^∗^	12	125.2 ± 8.9	169.7 ± 11.4	200.7 ± 13.7	233.7 ± 14.5	261.3 ± 19.0	284.4 ± 22.8	302.6 ± 26.0	316.3 ± 29.8	331.0 ± 30.8	341.1 ± 29.8	353.4 ± 33.6	359.1 ± 35.0	368.2 ± 36.1	373.5 ± 36.1
	Mid	12	122.1 ± 11.5	163.6 ± 13.8	189.5 ± 17.0	218.5 ± 17.5	239.1 ± 21.5	257.2 ± 22.4	269.0 ± 21.6	281.5 ± 23.8	292.0 ± 27.1	303.8 ± 24.1	309.2 ± 23.9	315.2 ± 25.3	320.9 ± 27.8	326.1 ± 28.1
	High	12	122.4 ± 5.4	163.0 ± 9.5	191.9 ± 11.4	221.6 ± 12.1	238.4 ± 16.5	255.2 ± 20.0	272.4 ± 21.2	287.2 ± 23.3	295.9 ± 23.5	302.4 ± 25.3	311.1 ± 28.6	317.8 ± 25.1	323.2 ± 28.2	327.0 ± 25.1

Male	Neg	12	127.4 ± 7.2	197.2 ± 9.1	259.5 ± 12.7	328.9 ± 19.1	382.8 ± 23.6	422.6 ± 29.1	460.6 ± 34.5	494.0 ± 35.3	518.9 ± 39.1	532.7 ± 38.3	551.7 ± 41.8	566.0 ± 42.6	582.6 ± 43.3	593.7 ± 43.9
	Low	12	128.1 ± 6.9	195.3 ± 8.5	256.2 ± 10.5	317.2 ± 13.0	369.5 ± 13.4	411.5 ± 13.8	447.5 ± 16.0	477.5 ± 17.9	501.7 ± 18.0	519.1 ± 19.2	534.6 ± 22.7	554.0 ± 25.5	568.2 ± 27.3	584.7 ± 28.5
	Mid^∗∗^	12	127.1 ± 10.2	192.2 ± 9.5	255.4 ± 10.6	315.9 ± 10.4	363.7 ± 13.5	397.1 ± 18.5	431.6 ± 24.4	454.4 ± 28.5	475.6 ± 33.9	489.9 ± 32.8	503.6 ± 36.5	517.6 ± 38.0	527.4 ± 43.4	540.4 ± 47.6
	High^∗∗∗^	12	127.8 ± 8.6	183.8 ± 13.1	240.7 ± 19.4	300.8 ± 22.4	343.2 ± 21.0	375.6 ± 24.0	414.8 ± 25.4	439.9 ± 28.2	456.9 ± 28.5	467.3 ± 28.7	481.0 ± 27.0	494.6 ± 30.6	509.7 ± 32.1	522.1 ± 34.2

*Note:* (1) Data are presented as the mean ± standard deviation. (2) A repeated‐measures ANOVA was used to statistically test the weekly body weight data of animals in each group. If the data do not meet the assumption of sphericity, a multivariate test (Pillai’s trace) is required. If there is a statistically significant difference in the group effect (dosage of the test substance), further pairwise comparisons are conducted using the least significant difference (LSD) method. The results of the significance test (compared with negative control, *α* = 0.05) are marked with asterisk (s).

^∗^ *p* < 0.05.

^∗∗^ *p* < 0.01.

^∗∗∗^ *p* < 0.001.

**TABLE 2:** Total body weight gain, feed consumption, food efficiency ratio, and average intake of D‐allulose.

Gender	Group	Total body weight gain (%)	Total feed consumption (g)	Total food efficiency ratio (g/100 g)	Average intake level (g/kg BW/day)
Female	Neg	177.5 ± 26.2 (*n* = 11)	2008.3 ± 214.2 (*n* = 7)	11.0 ± 1.1 (*n* = 7)	/
	Low	198.8 ± 27.0 (*n* = 12)	2157.5 ± 147.8 (*n* = 11)	11.5 ± 1.5 (*n* = 11)	2076 ± 202 (*n* = 11)
	Mid	168.0 ± 21.6 (*n* = 12)	1935.2 ± 191.1 (*n* = 11)	10.4 ± 0.8 (*n* = 11)	4145 ± 212 (*n* = 11)
	High	168.3 ± 30.8 (*n* = 12)	1929.8 ± 219.3 (*n* = 12)	10.6 ± 1.0 (*n* = 12)	8172 ± 651 (*n* = 12)

Male	Neg	366.6 ± 35.7 (*n* = 12)	2761.8 ± 210.4 (*n* = 12)	16.9 ± 1.2 (*n* = 12)	/
	Low	357.6 ± 29.5 (*n* = 12)	2648.5 ± 178.1 (*n* = 12)	17.3 ± 0.9 (*n* = 12)	1735 ± 73 (*n* = 12)
	Mid	327.2 ± 45.4^∗^ (*n* = 12)	2620.0 ± 228.8 (*n* = 12)	15.8 ± 1.0 (*n* = 12)	3600 ± 154 (*n* = 12)
	High	309.3 ± 25.5^∗∗^ (*n* = 12)	2428.7 ± 234.3^∗∗^ (*n* = 12)	16.3 ± 1.0 (*n* = 12)	6963 ± 351 (*n* = 12)

*Note:* (1) Data results are presented as means ± standard deviations, with *n* representing the number of animals. (2) One‐way ANOVA was used to test the total weight gain rate, total food intake, and total food utilization rate for animals of both sexes. If there are statistically significant indicators, further pairwise comparisons are conducted using the Bonferroni method. The results of the significance test (compared with negative control, *α* = 0.05) are marked with asterisk (s).

^∗^ *p* < 0.05.

^∗∗^ *p* < 0.01.

^∗∗∗^ *p* < 0.001.

The weekly feed consumption data for animals in each group are depicted in Table 3. Due to the gnawing behavior of rodents, some animals experienced severe feed loss, which was difficult to measure or estimate, resulting in significant individual variability and fluctuation in feed consumption data. Consequently, individuals with substantial bias were excluded. The ANOVA results demonstrated that the dosage and the duration of feeding significantly affected the feed consumption in both female and male animals (*p* < 0.05). Furthermore, pairwise comparisons of each level of the between‐group factor (i.e., each dosage group) indicated that the feed consumption of male animals in the high‐dose group was statistically different from those in the control group (*p* < 0.001). At the end of the experiment, the overall feed consumption for animals in each group is summarized in Table 2. Notably, the feed consumption of male animals in the high‐dose group was significantly lower when compared to the negative control group (*p* < 0.01). A dose‐dependent decrease was observed in total body weight gain and feed consumption.

**TABLE 3:** Feed consumption (g).

Gender	Group	*n*	Weeks												
			1	2	3	4	5	6	7	8	9	10	11	12	13
Female	Neg	7	189.5 ± 35.7	174.2 ± 32.2	148.8 ± 17.5	152.1 ± 16.2	161.3 ± 43.7	151.8 ± 22.8	164.0 ± 34.2	149.0 ± 16.7	145.2 ± 17.6	145.4 ± 13.8	146.6 ± 16.9	140.5 ± 13.7	140.0 ± 9.9
	Low	11	166.1 ± 36.6	196.3 ± 41.1	160.3 ± 12.5	169.6 ± 14.0	170.5 ± 18.3	169.9 ± 15.9	165.1 ± 21.8	158.6 ± 18.9	174.1 ± 15.6	157.2 ± 14.8	153.5 ± 12.4	157.7 ± 35.2	158.5 ± 12.6
	Mid	11	168.9 ± 46.7	155.2 ± 44.4	149.8 ± 14.6	155.5 ± 17.3	148.2 ± 23.0	138.7 ± 23.5	155.1 ± 20.1	148.0 ± 18.2	151.8 ± 12.9	142.6 ± 13.8	144.2 ± 17.6	134.4 ± 13.5	142.8 ± 11.8
	High	12	145.4 ± 34.6	167.6 ± 38.0	149.8 ± 20.0	155.4 ± 25.1	153.8 ± 28.0	152.5 ± 22.7	157.3 ± 24.7	140.7 ± 15.8	146.5 ± 15.4	142.4 ± 16.0	143.2 ± 12.7	132.7 ± 17.8	142.5 ± 14.0

Male	Neg	12	189.4 ± 30.8	217.2 ± 35.6	215.8 ± 14.3	226.1 ± 18.2	215.0 ± 20.3	217.2 ± 20.9	219.8 ± 12.7	219.2 ± 17.7	212.6 ± 19.3	209.4 ± 21.5	208.1 ± 18.4	204.3 ± 14.2	207.6 ± 19.7
	Low	12	186.7 ± 46.5	227.0 ± 43.8	193.5 ± 20.1	205.0 ± 18.9	202.5 ± 17.5	206.2 ± 17.8	207.5 ± 17.7	205.6 ± 10.2	199.2 ± 28.7	202.4 ± 14.0	205.0 ± 14.5	198.5 ± 13.4	209.6 ± 16.2
	Mid	12	186.0 ± 67.3	209.4 ± 43.3	203.5 ± 13.4	210.6 ± 11.4	202.1 ± 24.2	208.2 ± 18.5	202.7 ± 13.4	206.2 ± 25.9	206.0 ± 18.7	188.7 ± 28.1	201.1 ± 13.9	190.6 ± 18.8	205.0 ± 17.3
	High^∗∗∗^	12	165.1 ± 32.2	193.0 ± 24.6	188.7 ± 28.2	186.9 ± 27.2	186.1 ± 17.3	190.6 ± 19.7	196.4 ± 19.7	190.1 ± 17.9	182.6 ± 17.4	183.2 ± 16.8	186.4 ± 19.3	183.8 ± 19.1	195.8 ± 20.0

*Note:* (1) The data are presented as the mean ± standard deviation. (2) A repeated‐measures ANOVA was conducted to statistically test the weekly food intake data of animals in each group. If the data do not meet the assumption of sphericity, a multivariate test (Pillai’s trace) is required. If there is a statistically significant difference in the group effect (dosage of the test substance), further pairwise comparisons are made using the least significant difference (LSD) method. The results of the significance test (compared with negative control, *α* = 0.05) are marked with asterisk (s).

^∗∗∗^ *p* < 0.001.

The weekly food efficiency data for animals in each group are detailed in Table 4. Influenced by the feed consumption data, the food efficiency metric exhibited considerable individual variability and fluctuation, resulting in the elimination of data with significant bias. The ANOVA results showed that the duration of feeding significantly impacted the food efficiency data of both female and male animals (*p* < 0.05). However, the dosage of D‐allulose had no significant effect on the variation in weekly food efficiency. At the end of the experiment, the overall food efficiency for animals in each group is presented in Table 2. The ANOVA results revealed no statistically significant difference in the total food efficiency between the dosage groups and the negative control group.

**TABLE 4:** Food efficiency ratio (g/100 g).

Gender	Group	*n*	Weeks												
			1	2	3	4	5	6	7	8	9	10	11	12	13
Female	Neg	7	23.1 ± 5.6	17.9 ± 4.1	20.7 ± 6.9	14.3 ± 3.3	11.3 ± 4.0	10.2 ± 5.0	8.9 ± 4.9	7.9 ± 5.4	2.1 ± 2.8	7.1 ± 2.0	5.0 ± 4.8	7.0 ± 3.2	2.2 ± 3.1
	Low	11	27.8 ± 6.0	16.5 ± 3.4	20.0 ± 2.8	16.2 ± 4.0	13.4 ± 4.0	10.5 ± 4.4	7.9 ± 6.1	9.3 ± 4.6	5.7 ± 2.8	7.9 ± 3.9	3.5 ± 3.5	5.7 ± 4.3	3.9 ± 2.9
	Mid	11	25.2 ± 3.6	17.9 ± 5.8	19.4 ± 6.9	13.4 ± 4.2	12.2 ± 4.6	8.7 ± 2.5	7.4 ± 4.2	6.6 ± 4.0	8.1 ± 4.5	3.9 ± 3.6	3.3 ± 3.7	3.8 ± 5.5	3.4 ± 4.5
	High	12	29.2 ± 10.1	17.9 ± 5.1	20.1 ± 5.4	10.6 ± 4.7	11.1 ± 4.8	11.3 ± 4.3	9.3 ± 6.4	6.1 ± 4.3	4.4 ± 3.9	5.9 ± 3.3	4.9 ± 3.6	3.8 ± 4.1	2.7 ± 4.5

Male	Neg	12	37.6 ± 5.5	29.1 ± 4.1	32.1 ± 3.5	23.9 ± 3.0	18.4 ± 3.6	17.3 ± 4.3	15.2 ± 5.7	11.3 ± 2.7	6.4 ± 2.0	9.0 ± 2.8	6.9 ± 1.9	8.1 ± 2.5	5.3 ± 2.5
	Low	12	37.7 ± 8.1	27.7 ± 5.7	31.6 ± 2.4	25.6 ± 3.1	20.9 ± 1.5	17.5 ± 3.0	14.4 ± 2.2	11.8 ± 1.8	8.6 ± 2.0	7.7 ± 3.3	9.4 ± 2.7	7.0 ± 2.4	7.8 ± 3.4
	Mid	12	37.4 ± 8.9	31.2 ± 5.7	29.8 ± 2.7	22.6 ± 3.0	16.8 ± 4.4	16.4 ± 2.7	11.2 ± 3.2	10.1 ± 3.2	6.9 ± 3.6	7.2 ± 2.9	6.9 ± 1.2	4.9 ± 4.0	6.2 ± 3.1
	High	12	34.6 ± 6.7	29.7 ± 3.9	32.3 ± 4.9	23.3 ± 5.6	17.3 ± 3.9	20.6 ± 4.3	12.7 ± 3.3	9.0 ± 2.8	5.6 ± 3.8	7.5 ± 3.7	7.2 ± 4.3	8.3 ± 2.7	6.3 ± 2.4

*Note:* (1) Data are presented as the mean ± standard deviation. (2) A repeated‐measures ANOVA was used to statistically test the weekly food utilization data of animals in each group. If the data do not meet the assumption of sphericity, a multivariate test (Pillai’s trace) is required. If there is a statistically significant difference in the group effect (dosage of the test substance), further pairwise comparisons are conducted using the least significant difference (LSD) method. No statistically significant differences were observed among all variables in this table.

### 3.3. Ophthalmic Examination

All animals underwent ophthalmic examinations, including the cornea, iris, conjunctiva, and lens, during the environmental adaptation/quarantine period and postexperiment, and no abnormalities were found.

### 3.4. Urine Analysis

Urine samples from animals in each group were collected at the end of the experiment for routine urinalysis, and the results are presented in Table 5. There were no anomalies in the routine urinalysis parameters for animals in any group, and no statistical differences were found across groups.

**TABLE 5:** Urine examination (number of animals).

Gender	Group	APP		pH			SG			GLU		KET			BLD				PRO				BIL			URO		NIT		LEU	
		Normal	Abnormal	≤ 5.5	6.0–8.0	≥ 8.5	≤ 1.005	1.010–1.025	≥ 1.030	−Or ±	1+∼3+	− Or ±	1+	2+∼3 +	−Or ±	1 +	2 +	3 +	−Or ±	1 +	2 +	3 +	—	1 +	2+∼3+	3.2	≥ 16	—	+	−Or ±	1+∼3+
Female	Neg	11	0	0	11	0	0	10	1	11	0	11	0	0	11	0	0	0	11	0	0	0	11	0	0	11	0	9	2	11	0
	Low	12	0	0	12	0	1	10	1	12	0	12	0	0	12	0	0	0	12	0	0	0	12	0	0	12	0	10	2	12	0
	Mid	12	0	0	12	0	0	10	2	12	0	12	0	0	12	0	0	0	10	2	0	0	12	0	0	12	0	10	2	12	0
	High	12	0	0	12	0	1	11	0	12	0	12	0	0	12	0	0	0	12	0	0	0	12	0	0	12	0	9	3	12	0

Male	Neg	12	0	0	11	1	0	11	1	12	0	11	1	0	11	0	1	0	10	1	1	0	11	1	0	12	0	11	1	11	1
	Low	12	0	0	12	0	0	11	1	12	0	12	0	0	11	1	0	0	9	3	0	0	12	0	0	12	0	12	0	11	1
	Mid	12	0	0	12	0	1	11	0	12	0	12	0	0	12	0	0	0	9	3	0	0	12	0	0	12	0	11	1	11	1
	High	12	0	0	12	0	0	10	2	12	0	10	2	0	9	3	0	0	8	3	1	0	12	0	0	12	0	12	0	12	0

*Note:* (1) APP: Appearance, GLU: Glucose in Urine, KET: Ketone Bodies in Urine, BLD: Occult Blood, PRO: Protein in Urine, BIL: Bilirubin in Urine, URO: Urobilinogen in Urine, NIT: Nitrite, LEU: Leukocytes. (2) “—“indicates a negative result; “+/−” indicates a trace amount result. (3) Data were statistically analyzed using the Kruskal–Wallis rank sum test.

Abbreviation: SG: Specific Gravity.

### 3.5. Hematological Assessment

Hematological analyses were performed using whole blood samples collected from animals in all groups. These analyses included a complete blood count with differential white blood cell (WBC) analysis, red blood cell (RBC) enumeration, HGB concentration, HCT measurement, PLT count, and coagulation profiles including PT and APTT. The data are tabulated in Table 6. The mean values of these hematological parameters for both genders within each group were found to be within the historical reference ranges of our laboratory. Male animals in all dosage groups showed a statistically significant decrease in HGB and HCT in comparison with the negative control, with a discernible dose–response relationship. Specifically, the mean HCT in mid (*p* < 0.05)‐ and high‐dose (*p* < 0.001) males was significantly lower than the negative control, and the HGB in the high‐dose males was significantly lower than the negative control (*p* < 0.001). Additionally, the mean PLT count in low‐ and mid‐dose females as well as mid‐ and high‐dose males was significantly elevated compared to the negative control, reaching statistical significance (*p* < 0.01).

**TABLE 6:** Hematology assessment.

Gender	Group	*n*	WBC (10^9^/L)	MONO%	LYMPH%	NEUT%	EO%	RBC (10^12^/L)	HGB (g/L)	HCT%	PLT (10^9^/L)	PT (s)	APTT (s)
Female	Neg	11	2.39 ± 0.61	3.4 ± 1.0	73.3 ± 6.1	21.9 ± 6.6	1.4 ± 0.5	7.50 ± 0.33	133 ± 6	39.3 ± 1.8	975 ± 78	7.9 ± 0.5	12.7 ± 1.2
	Low	12	2.34 ± 0.92	3.8 ± 1.0	73.8 ± 8.1	19.8 ± 7.4	2.5 ± 0.5^∗∗^	7.42 ± 0.37	132 ± 5	38.2 ± 1.3	1199 ± 181^∗∗^	7.6 ± 0.3	11.9 ± 1.8
	Mid	12	3.20 ± 0.81	2.9 ± 1.1	78.7 ± 7.6	17.3 ± 6.6	1.2 ± 0.6	7.48 ± 0.26	133 ± 5	38.4 ± 1.5	1217 ± 96^∗∗∗^	7.5 ± 0.3	13.5 ± 0.9
	High	12	2.62 ± 1.10	2.2 ± 0.6^∗^	80.2 ± 5.9	16.0 ± 5.4	1.6 ± 0.9	7.44 ± 0.26	129 ± 4	37.9 ± 1.2	1099 ± 145	7.6 ± 0.4	13.1 ± 2.0

Male	Neg	12	4.74 ± 1.44	2.8 ± 1.4	68.3 ± 9.9	27.4 ± 8.6	1.6 ± 0.8	8.29 ± 0.27	141 ± 5	41.7 ± 1.7	1129 ± 108	8.0 ± 0.5	14.6 ± 1.9
	Low	12	4.07 ± 0.86	3.1 ± 1.1	68.2 ± 10.5	27.0 ± 9.6	1.6 ± 1.0	8.55 ± 0.40	141 ± 4	40.7 ± 1.3	1255 ± 102	7.9 ± 0.2	15.1 ± 1.0
	Mid	12	4.60 ± 1.29	3.4 ± 1.1	66.2 ± 9.1	29.4 ± 8.3	1.1 ± 0.6	8.62 ± 0.26	138 ± 6	39.7 ± 1.9^∗^	1449 ± 162^∗∗∗^	7.9 ± 0.3	14.0 ± 1.6
	High	12	4.27 ± 1.97	2.8 ± 0.9	66.5 ± 6.9	29.3 ± 6.8	1.4 ± 1.1	8.51 ± 0.49	128 ± 6^∗∗∗^	37.5 ± 1.9^∗∗∗^	1380 ± 192^∗∗^	8.0 ± 0.5	14.3 ± 1.6

*Note:* (1) Results are presented as means ± standard deviations, with *n* representing the number of animals. (2) MONO: Monocytes, LYMPH: Lymphocytes, NEUT: Neutrophils, EO: Eosinophils, HGB: Hemoglobin, HCT: Hematocrit, PLT: Platelets, PT: Prothrombin Time. (3) Basophils were not detected in animals of all groups, hence not displayed in this table. (4) Hematological parameter data for animals of both sexes were analyzed using one‐way ANOVA. Data not meeting the assumption of homogeneity of variance were corrected using Welch’s test; if statistical differences were found, further pairwise comparisons were conducted using the Bonferroni method (for homogeneity of variance) or the Games–Howell method (for heterogeneity of variance). The results with statistical significance were marked with asterisk (s).

Abbreviations: APTT: Activated Partial Thromboplastin Time, RBC: Red Blood Cells, WBC: White Blood Cells.

^∗^ *p* < 0.05.

^∗∗^ *p* < 0.01.

^∗∗∗^ *p* < 0.001.

### 3.6. Serum Biochemical Analysis

The effects of D‐allulose on serum clinical biochemical parameters in female and male rats are presented in Table 7. Alterations in biochemical metrics differed between female and male rats following D‐allulose exposure, with dose‐dependent trends observed for certain metrics when compared to the control group.

**TABLE 7:** Clinical biochemistry assessment.

Gender	Group	*n*	ALT (U/L)	AST (U/L)	ALP (U/L)	TP (g/L)	Alb (g/L)	Glu (mmol/L)	TG (mmol/L)	TC (mmol/L)	HDL‐C (mmol/L)	LDL‐C (mmol/L)	Cr (umol/L)	Urea (mmol/L)	K^+^ (mmol/L)	Na^+^ (mmol/L)	Cl^+^ (mmol/L)
Female	Neg	11	27.6 ± 5.4	103.4 ± 16.3	40 ± 10	61.4 ± 5.3	47.2 ± 5.2	7.96 ± 0.71	0.27 ± 0.19	1.90 ± 0.67	1.02 ± 0.36	0.37 ± 0.16	36.6 ± 2.8	6.59 ± 1.14	4.5 ± 0.2	152 ± 2	109 ± 2
	Low	12	27.0 ± 5.2	121.5 ± 35.2	40 ± 12	63.8 ± 5.5	48.9 ± 5.5	6.75 ± 0.83^∗∗∗^	0.50 ± 0.22^∗^	1.67 ± 0.24	0.97 ± 0.15	0.29 ± 0.08	35.9 ± 4.5	6.53 ± 0.96	4.6 ± 0.3	150 ± 2^∗^	108 ± 2
	Mid	12	28.4 ± 16.9	108.0 ± 44.9	42 ± 10	64.6 ± 5.6	48.9 ± 4.1	5.86 ± 0.47^∗∗∗^	0.33 ± 0.11	1.67 ± 0.40	0.98 ± 0.24	0.26 ± 0.09	30.9 ± 1.8^∗∗∗^	7.15 ± 0.95	4.6 ± 0.2	152 ± 2	110 ± 2
	High	12	22.5 ± 4.8	99.2 ± 15.7	57 ± 12^∗∗^	60.5 ± 4.8	46.2 ± 4.7	7.19 ± 0.73	0.31 ± 0.14	1.39 ± 0.30	0.76 ± 0.14	0.22 ± 0.09^∗∗^	30.4 ± 3.5^∗∗∗^	6.48 ± 0.78	4.8 ± 0.5	152 ± 1	110 ± 3

Male	Neg	12	32.8 ± 6.2	131.1 ± 21.9	77 ± 19	58.2 ± 2.2	40.9 ± 1.5	7.69 ± 0.71	0.39 ± 0.25	1.47 ± 0.37	0.76 ± 0.18	0.38 ± 0.15	28.2 ± 2.4	5.72 ± 0.80	5.2 ± 0.3	155 ± 2	108 ± 1
	Low	12	33.4 ± 12.3	130.5 ± 21.2	73 ± 12	60.9 ± 1.7^∗^	41.4 ± 1.3	7.38 ± 0.80	0.43 ± 0.20	1.90 ± 0.44^∗^	0.96 ± 0.22	0.60 ± 0.20^∗^	29.2 ± 2.9	6.25 ± 0.83	4.7 ± 0.2^∗∗^	153 ± 1	109 ± 1
	Mid	12	35.8 ± 8.1	148.2 ± 33.0	107 ± 29^∗^	60.2 ± 1.4	41.9 ± 1.2	7.17 ± 0.41	0.29 ± 0.06	1.97 ± 0.29^∗∗^	1.03 ± 0.16^∗∗^	0.65 ± 0.19^∗∗^	27.8 ± 3.9	6.65 ± 0.84	4.8 ± 0.3^∗^	154 ± 1	110 ± 2
	High	12	30.5 ± 5.4	184.7 ± 33.5^∗∗∗^	171 ± 44^∗∗∗^	58.9 ± 3.6	43.8 ± 2.3^∗∗^	8.27 ± 1.12	0.34 ± 0.21	1.97 ± 0.37^∗^	0.94 ± 0.19	0.69 ± 0.14^∗∗∗^	27.5 ± 5.8	6.91 ± 1.16^∗^	5.4 ± 0.5	152 ± 2^∗∗^	109 ± 2

*Note:* (1) Results are presented as means ± standard deviations, with *n* representing the number of animals. (2) ALT: Alanine Aminotransferase, AST: Aspartate Aminotransferase, ALP: Alkaline Phosphatase, Alb: Albumin, Glu: Glucose, TG: Triglycerides, HDL‐C: High‐Density Lipoprotein Cholesterol, LDL‐C: Low‐Density Lipoprotein Cholesterol, Cr: Creatinine, Urea: Urea. (3) The level of Gamma‐Glutamyl Transferase (GGT) did not reach the detection limit, and the data for this parameter are not listed. (4) Hematological parameters for animals of both sexes were analyzed using one‐way ANOVA. Data not meeting the assumption of homogeneity of variance were corrected using Welch’s test; if statistical differences were found, further pairwise comparisons were conducted using the Bonferroni method (for homogeneity of variance) or the Games–Howell method (for heterogeneity of variance). The results with statistical significance were marked with asterisk (s).

Abbreviations: TC: Total Cholesterol, TP: Total Protein.

^∗^ *p* < 0.05.

^∗∗^ *p* < 0.01.

^∗∗∗^ *p* < 0.001.

For female animals, no significant differences in ALT and AST levels were observed among all female groups, indicating no obvious D‐allulose‐induced hepatic injury in female rats. ALP activity was significantly elevated in the high‐dose group (57 ± 12 U/L, *p* < 0.01) relative to the control group (40 ± 10 U/L), while no differences were found in the low‐ and mid‐dose groups. TP and Alb concentrations remained unchanged across all female groups, suggesting that D‐allulose had no effect on protein synthesis and metabolism in female rats.

For glucose and lipid metabolism metrics in female animals, D‐allulose exposure induced statistically significant decrease in blood glucose in female rats of low (6.75 ± 0.83 mmol/L, *p* < 0.001)‐ and mid‐dose (5.86 ± 0.47 mmol/L, *p* < 0.001) groups compared with the control group (7.96 ± 0.71 mmol/L), while no significant difference was noted in the high‐dose group. TG levels were only increased in the low‐dose group (0.50 ± 0.22 mmol/L, *p* < 0.05), with no statistical differences in the mid‐ and high‐dose groups. TC showed a decreasing trend with increasing D‐allulose dose, and LDL‐C was significantly reduced in the high‐dose group (0.22 ± 0.09 mmol/L, *p* < 0.01) relative to the control group (0.37 ± 0.16 mmol/L); no significant changes in HDL‐C were observed in any D‐allulose group.

Regarding renal function metrics in female animals, Cr levels were slightly but significantly lower in the mid (30.9 ± 1.8 μmol/L, *p* < 0.001)‐ and high‐dose (30.4 ± 3.5 μmol/L, *p* < 0.001) groups compared with the control group (36.6 ± 2.8 μmol/L), whereas urea concentrations did not differ significantly across all female groups. For electrolyte profiles, only sodium (Na^+^) levels in the low‐dose group (150 ± 2 mmol/L, *p* < 0.05) were slightly decreased relative to the control group, while potassium (K^+^) and chloride (Cl^−^) concentrations remained unaltered in all D‐allulose‐treated groups. These results indicated that D‐allulose exerted a minimal impact on renal function and electrolyte homeostasis in female rats.

In contrast to female animals, D‐allulose exposure induced more pronounced alterations in hepatic function metrics in male rats with a dose‐dependent trend. ALT levels showed no statistical differences among all male groups (*p* > 0.05), while AST activity was elevated in the high‐dose group (184.7 ± 33.5 U/L, *p* < 0.001) compared with the control group (131.1 ± 21.9 U/L). ALP activity was significantly increased in the mid (107 ± 29 U/L, *p* < 0.05)‐ and high‐dose (171 ± 44 U/L, *p* < 0.001) groups, presenting a clear dose‐related increase. TP concentration was only slightly elevated in the low‐dose group (60.9 ± 1.7 g/L, *p* < 0.05), and Alb levels were slightly higher in the high‐dose group (43.8 ± 2.3 g/L, *p* < 0.01) relative to the control group (40.9 ± 1.5 g/L).

Glu levels in male animals showed no significant differences across all groups, indicating the absence of a hypoglycemic effect of D‐allulose in male animals. For lipid metabolism metrics, TG levels remained unchanged in all D‐allulose groups, while TC levels were significantly increased in the low (*p* < 0.05)‐, mid (*p* < 0.01)‐, and high‐dose (*p* < 0.05) groups compared with the control group. HDL‐C levels were elevated in the mid‐dose group (1.03 ± 0.16 mmol/L, *p* < 0.01), and LDL‐C levels were significantly increased in the low (*p* < 0.05), mid (*p* < 0.01)‐, and high‐dose (*p* < 0.001) groups with a dose‐dependent trend, suggesting that D‐allulose disturbed cholesterol metabolism in male animals.

Renal function metrics (Cr and urea) in male animals showed no significant differences in Cr levels across all groups, while the urea concentration was only elevated in the high‐dose group (6.91 ± 1.16 mmol/L, *p* < 0.05). For electrolytes, K^+^ levels were mildly decreased in the low (*p* < 0.01)‐ and mid‐dose (*p* < 0.05) groups, and Na^+^ levels were slightly lower in the high‐dose group (152 ± 2 mmol/L, *p* < 0.01); Cl^−^ levels remained unaltered in all D‐allulose groups (*p* > 0.05).

### 3.7. Pathological Examination

#### 3.7.1. Gross Anatomical Observations and Organ Weights

Upon completion of the experiment, a gross dissection of the animals was performed. No macroscopic abnormalities were observed in the organs and tissues across all groups. The absolute and relative organ weights (organ‐to‐body weight ratio and organ‐to‐brain weight ratio) are presented in Tables 8, 9, and 10.

**TABLE 8:** Absolute weight of organs (g).

Gender	Group	*n*	Fasting body weight	Brain	Heart	Thymus	Adrenal gland	Liver	Kidney	Spleen	Ovary/testis	Uterus/epididymis
Female	Neg	11	311.49 ± 36.90	1.93 ± 0.13	10.49 ± 0.95	0.42 ± 0.10	0.09 ± 0.02	8.39 ± 1.15	2.10 ± 0.20	0.61 ± 0.11	0.20 ± 0.04	0.74 ± 0.22
	Low	12	352.49 ± 36.34^∗^	1.89 ± 0.17	11.22 ± 1.37	0.55 ± 0.17	0.09 ± 0.01	9.53 ± 1.18	2.33 ± 0.26	0.64 ± 0.09	0.27 ± 0.25	0.65 ± 0.19
	Mid	12	306.20 ± 25.28	1.92 ± 0.24	10.09 ± 1.08	0.41 ± 0.11	0.11 ± 0.03	9.21 ± 1.37	2.38 ± 0.20^∗^	0.61 ± 0.12	0.19 ± 0.03	0.71 ± 0.17
	High	12	304.20 ± 23.66	1.98 ± 0.17	10.23 ± 1.06	0.40 ± 0.12	0.10 ± 0.02	9.25 ± 0.81	2.52 ± 0.22^∗∗∗^	0.64 ± 0.09	0.19 ± 0.03	0.94 ± 0.29

Male	Neg	12	564.92 ± 42.21	2.12 ± 0.17	17.01 ± 1.84	0.66 ± 0.23	0.09 ± 0.02	14.98 ± 2.07	3.75 ± 0.60	1.04 ± 0.27	3.72 ± 0.31	1.75 ± 0.35
	Low	12	551.29 ± 30.39	2.18 ± 0.08	16.58 ± 1.84	0.56 ± 0.11	0.10 ± 0.03	15.82 ± 1.67	4.09 ± 0.36	0.91 ± 0.13	3.74 ± 0.36	1.71 ± 0.22
	Mid	12	509.04 ± 42.33^∗∗^	2.10 ± 0.13	15.73 ± 1.11	0.49 ± 0.11	0.08 ± 0.01	14.93 ± 1.59	4.28 ± 0.42	0.90 ± 0.13	3.61 ± 0.45	1.78 ± 0.23
	High	12	486.78 ± 33.24^∗∗∗^	2.13 ± 0.18	15.94 ± 2.76	0.57 ± 0.20	0.10 ± 0.02	15.12 ± 3.05	4.15 ± 0.78	0.89 ± 0.12	3.75 ± 0.27	1.87 ± 0.46

*Note:* (1) Results are presented as means ± standard deviations, with *n* representing the number of animals. (2) A one‐way ANOVA was conducted for the absolute organ weights of animals of both sexes. Data not meeting the assumption of homogeneity of variance were corrected using Welch’s test; if statistical differences were found, further pairwise comparisons were conducted using the Bonferroni method (for homogeneity of variance) or the Games–Howell method (for heterogeneity of variance). The results with statistical significance were marked with asterisk (s).

^∗^ *p* < 0.05.

^∗∗^ *p* < 0.01.

^∗∗∗^ *p* < 0.001.

**TABLE 9:** Relative weight of organs (organ vs. body weight, × 10^−2^).

Gender	Group	*n*	Brain	Heart	Thymus	Adrenal gland	Liver	Kidney	Spleen	Ovary/testis	Uterus/epididymis
Female	Neg	11	0.625 ± 0.061	3.393 ± 0.342	0.135 ± 0.029	0.031 ± 0.011	2.697 ± 0.242	0.678 ± 0.059	0.200 ± 0.049	0.065 ± 0.015	0.242 ± 0.083
	Low	12	0.542 ± 0.084	3.181 ± 0.196	0.157 ± 0.046	0.027 ± 0.004	2.711 ± 0.263	0.662 ± 0.054	0.183 ± 0.024	0.076 ± 0.069	0.187 ± 0.057
	Mid	12	0.632 ± 0.101	3.306 ± 0.340	0.135 ± 0.037	0.036 ± 0.010	3.006 ± 0.356^∗^	0.779 ± 0.057^∗∗∗^	0.200 ± 0.036	0.063 ± 0.010	0.232 ± 0.050
	High	12	0.653 ± 0.070	3.376 ± 0.414	0.129 ± 0.033	0.034 ± 0.006	3.045 ± 0.190^∗^	0.829 ± 0.055^∗∗∗^	0.210 ± 0.032	0.062 ± 0.010	0.307 ± 0.082

Male	Neg	12	0.376 ± 0.032	3.027 ± 0.416	0.116 ± 0.040	0.016 ± 0.004	2.644 ± 0.212	0.666 ± 0.105	0.184 ± 0.038	0.664 ± 0.066	0.310 ± 0.059
	Low	12	0.397 ± 0.032	3.009 ± 0.319	0.101 ± 0.020	0.019 ± 0.005	2.864 ± 0.190	0.744 ± 0.067	0.165 ± 0.021	0.681 ± 0.072	0.309 ± 0.031
	Mid	12	0.415 ± 0.040	3.100 ± 0.245	0.096 ± 0.026	0.016 ± 0.002	2.934 ± 0.231	0.846 ± 0.106^∗∗^	0.177 ± 0.023	0.713 ± 0.109	0.350 ± 0.040
	High	12	0.438 ± 0.033	3.260 ± 0.381	0.116 ± 0.037	0.020 ± 0.004	3.106 ± 0.591^∗^	0.855 ± 0.156^∗∗^	0.184 ± 0.023	0.773 ± 0.068^∗^	0.383 ± 0.076

*Note:* (1) Results are presented as means ± standard deviations. (2) A one‐way ANOVA was conducted for the relative organ weights (organ‐to‐body ratio) of animals of both sexes. Data not meeting the assumption of homogeneity of variance were corrected using Welch’s test; if statistical differences were found, further pairwise comparisons were conducted using the Bonferroni method (for homogeneity of variance) or the Games–Howell method (for heterogeneity of variance). Significance was tested at *α* = 0.05, and results were marked with asterisk (s).

^∗^ *p* < 0.05.

^∗∗^ *p* < 0.01.

^∗∗∗^ *p* < 0.001.

**TABLE 10:** Relative weight of organs (organ vs. brain).

Gender	Group	*n*	Heart	Thymus	Adrenal gland	Liver	Kidney	Spleen	Ovary/testis	Uterus/epididymis
Female	Neg	11	2.845 ± 0.439	0.114 ± 0.033	0.026 ± 0.008	2.264 ± 0.330	0.571 ± 0.101	0.168 ± 0.049	0.055 ± 0.016	0.205 ± 0.084
	Low	12	3.221 ± 0.720	0.158 ± 0.055	0.028 ± 0.005	2.759 ± 0.761	0.666 ± 0.130	0.185 ± 0.045	0.083 ± 0.093	0.188 ± 0.067
	Mid	12	2.845 ± 0.759	0.120 ± 0.059	0.031 ± 0.008	2.605 ± 0.791	0.678 ± 0.210	0.171 ± 0.045	0.055 ± 0.018	0.199 ± 0.059
	High	12	2.669 ± 0.551	0.103 ± 0.034	0.028 ± 0.009	2.423 ± 0.535	0.659 ± 0.139	0.164 ± 0.024	0.049 ± 0.014	0.248 ± 0.093

Male	Neg	12	3.881 ± 0.880	0.144 ± 0.040	0.021 ± 0.007	3.390 ± 0.663	0.861 ± 0.222	0.235 ± 0.063	0.871 ± 0.188	0.399 ± 0.108
	Low	12	3.502 ± 0.521	0.117 ± 0.027	0.022 ± 0.006	3.340 ± 0.454	0.863 ± 0.095	0.193 ± 0.032	0.791 ± 0.106	0.360 ± 0.060
	Mid	12	3.588 ± 0.328	0.112 ± 0.030	0.019 ± 0.003	3.421 ± 0.552	0.978 ± 0.126	0.206 ± 0.043	0.826 ± 0.138	0.405 ± 0.048
	High	12	3.525 ± 0.524	0.126 ± 0.045	0.021 ± 0.006	3.344 ± 0.706	0.931 ± 0.216	0.199 ± 0.033	0.842 ± 0.152	0.416 ± 0.101

*Note:* (1) Results are presented as means ± standard deviations. (2) A one‐way ANOVA was conducted for the relative organ weights (organ‐to‐brain ratio) of animals of both sexes. Data not meeting the assumption of homogeneity of variance were corrected using Welch’s test; if statistical differences were found, further pairwise comparisons were conducted using the Bonferroni method (for homogeneity of variance) or the Games–Howell method (for heterogeneity of variance). Significance was tested at *α* = 0.05, and the results were marked with asterisk (s).

^∗^ *p* < 0.05.

^∗∗^ *p* < 0.01.

^∗∗∗^ *p* < 0.001.

In female rats, no significant differences were observed in the absolute weights of most organs except the kidney compared with the negative control group: the absolute kidney weight was significantly increased in the mid (*p* < 0.05)‐ and high‐dose group (*p* < 0.001), showing a dose‐dependent upward trend. For organ‐to‐body weight ratios, the liver (*p* < 0.05) and kidney (^∗∗∗^ *p* < 0.001) ratios in the mid and high‐dose groups were significantly increased, consistent with the change trend of absolute kidney weight; other organ ratios showed no statistical differences. Notably, all organ‐to‐brain weight ratios in female rats exhibited no significant intergroup differences, indicating that the relative proportion of each organ‐to‐brain tissue remained unchanged.

In male rats, all detected organs showed no significant differences in absolute weights among all dose groups relative to the negative control. Notably, the body weight of male rats decreased significantly in the mid‐dose (*p* < 0.01) and high‐dose (*p* < 0.001) groups, which may partially contribute to the elevation of organ‐to‐body weight ratios in relevant organs. For organ‐to‐body weight ratios, the kidney ratios in the mid‐ and high‐dose groups were significantly increased (*p* < 0.01) in a dose‐dependent manner; the liver and testis ratios in the high‐dose group were significantly elevated (*p* < 0.05); no significant differences were found in the ratios of brain, heart, thymus, adrenal gland, and spleen. Consistent with female rats, all organ‐to‐brain weight ratios in male rats exhibited no significant intergroup differences, with no statistically significant changes observed in all organ‐to‐brain weight ratios across all groups.

#### 3.7.2. Histopathological Examination

Histopathological examinations were conducted on the brain, cerebellum, spinal cord, pituitary gland, heart, liver, spleen, lungs, kidneys, adrenal glands, thyroid gland, thymus, lymph nodes, pancreas, esophagus, stomach, duodenum, jejunum, ileum, cecum, colon, rectum, bladder, salivary glands, eyeballs, skeletal muscles, sciatic nerves, bone joints, bone marrow, testes, epididymides, prostate, uteri, and ovaries of rats in high‐dose and negative control groups. No lesions that were directly related to the D‐allulose intake were found. The detailed pathological descriptions of each organ or tissue are provided in the Supporting information (Supporting information: Supporting Results S2).

## 4. Discussion

### 4.1. Core Findings and Preliminary Interpretation of the 90‐Day Oral Toxicity Study

The present 90‐day feeding study was designed to evaluate the subchronic toxicity of D‐allulose produced by a newly developed one‐step fermentation process employing GM *Escherichia coli* AS10. Overall, no mortality or treatment‐related clinical signs were observed, and no histopathological lesions attributable to the test substance were detected. The most statistically significant alterations observed in the present study—namely, reduced body weight gain [37, 38], diminished feed consumption [37], elevated absolute kidney weight [38, 39], decreased HCT and HGB [38], increased PLT [39], and rises in serum ALP [38] and TC [38]—have been reported previously. Among these, the alternations in HCT, HGB, PLT, and TC were modest or remained within our historical reference value range, whereas the reductions in body weight gain and feed consumption, the increase in absolute kidney weight, and the elevation in serum ALP merit closer attention.

D‐Allulose exposure induced a moderate, statistically significant and dose‐dependent reduction in body weight gain in male rats. Notably, this weight change was not accompanied by any treatment‐related and toxicologically relevant abnormalities, including clinical signs, histopathological lesions, hematological/biochemical toxic markers (e.g., liver/kidney function impairment, electrolyte disturbance). In toxicological assessment, a single parametric change without concurrent adverse effects is insufficient to be classified as a toxic response. Consistent with this principle, the observed body weight gain reduction in male rats cannot be deemed a toxicological effect. Furthermore, numerous previous studies have consistently validated that D‐allulose possesses well‐documented weight‐modulating pharmacological or health‐promoting effects, particularly in the context of energy metabolism regulation and obesity management [37, 38, 40]. The underlying mechanisms contributing to the weight change may involve four complementary metabolic regulatory pathways, as elaborated below: First, D‐allulose competes with D‐glucose and D‐fructose for the apical transporters SGLT1, GLUT2 [41], and, more prominently, GLUT5—a facilitative transporter that mediates fructose uptake [2]—while also transiently inhibiting *α*‐amylase‐mediated starch hydrolysis [42]; together, these effects curtail net carbohydrates absorption and therefore reduce the caloric yield. Second, rapid luminal appearance of allulose evokes a robust GLP‐1 response, and the ensuing vagal signaling is known to acutely suppress appetite and, over weeks, to lower cumulative energy intake [40]. Third, allulose that escapes small‐intestinal uptake is fermented to short‐chain fatty acids in the large bowel, which not only amplify GLP‐1 release but also enhance lipid oxidation and reduce adiposity [43, 44]. In addition to the aforementioned mechanisms, the inherent low caloric value of D‐allulose directly reduces metabolizable energy density. Generally, the effect in reducing body weight is regarded as a positive efficacy of D‐allulose, particularly in the context of obesity management. Consequently, we propose that such effects of D‐allulose should be attributed to pharmacological activity rather than toxicity, given the absence of accompanying adverse clinical signs or histopathological lesions.

A concomitant decrease in feed consumption was observed among male rats receiving the 10% (w/w) D‐allulose diet. Beyond the GLP‐1–dependent satiety as discussed above, the palatability alternation might also contribute to this effect. However, a preliminary in‐house human sensory trial detected no off‐flavor or cloying sweetness in D‐allulose at concentration up to 36% (w/v). Furthermore, an additional two positive control groups receiving weight‐matched incorporation (10%, w/w) of D‐fructose or sucrose, sweeteners with only a minor flavor difference from D‐allulose, failed to suppress food intake in male rats (unpublished data), indicating that palatability is unlikely to explain the observed reduction in feed consumption. Additionally, in a separate study [37], D‐allulose at a lower incorporation level (3%) also reduced feed intake in rodents, further supporting that palatability may not be the primary contributor to the effect.

Absolute kidney weight was modestly elevated in female animals of mid‐ and high‐dose groups (Table 8), which is in line with observations in other studies [38, 39]. Toxicokinetic data demonstrate that most of absorbed allulose is renally excreted unchanged within 24 h in rodents [45]. When calculating the mean urinary concentrations of allulose in humans at an ingested dose of 10–40 g per day from enriched food and drinks, D‐allulose levels in urine may easily exceed 10 mM [46]. Such concentrations impose a chronic osmotic load on the renal medulla, analogous to that described for high‐salt diets, which induce tubular hypertrophy, glomerular hyperfiltration, and temporarily increased kidney weight [47]. Our findings support the interpretation that the observed weight gain of kidneys represents an adaptive, nonadverse response to sustained osmotic load rather than intrinsic nephrotoxicity.

A moderate elevation in serum ALP levels was observed in male rats of the mid‐dose group, whereas a more pronounced increase (up to 122%) was noted in those of the high‐dose group, with values exceeding our laboratory’s historical reference range. Elevated ALP levels are typically associated with liver and bone disorders [48]; however, no corresponding pathological changes were observed in this study, suggesting that ALP may derive from alternative sources. Beyond the liver and bones, the intestine constitutes a non‐negligible source of serum ALP. It has been reported that intestinal mucosal epithelial cells can secrete substantial quantities of ALP upon the consumption of large amounts of high‐fat food, with most being secreted luminally and a minor fraction entering the circulation [48]. In fact, various dietary components may influence the activity and expression of intestinal ALP [49]. Consequently, we hypothesize that allulose may directly or indirectly stimulate ALP secretion through a similar mechanism. In any case, given the absence of supporting histopathological evidence, the elevation in ALP cannot be ascribed to toxicological significance. This finding is therefore deemed a minor, nonadverse effect.

Several limitations of the present study merit consideration. First, despite preliminary sensory evaluations and comparisons with the other two sweetener controls indicating a low likelihood that palatability accounts for the reduced food intake in male rats, the potential confounding effect of palatability on ingestive behavior cannot be entirely excluded. Second, the absence of a recovery phase precludes the assessment of whether altered parameters (e.g., serum ALP levels) might normalize following the discontinuation of D‐allulose exposure. Furthermore, for certain parametric changes, such as the elevated serum ALP in high‐dose male rats, while histopathological correlates were not observed, the underlying molecular mechanisms and physiological implications remain incompletely elucidated, limiting definitive confirmation of their nontoxicological nature. These constraints underscore the necessity for further research to resolve such uncertainties.

### 4.2. Comparative Analysis With Previous 90‐Day Oral Toxicity Studies of D‐Allulose

Our 90‐day dietary toxicity study aligns with two key previous investigations [38, 39] in confirming the low toxicological profile of D‐allulose, while extending the evidence base through a unique study design and novel production context. Consistent with these prior studies, no treatment‐related mortality or overt toxicity was observed, and common physiological responses, including reduced body weight gain in male rats [38] and increased absolute/relative kidney weights [38, 39], were replicated, with no corresponding histopathological lesions to indicate intrinsic organ toxicity. These congruent findings collectively validate that D‐allulose, regardless of production method, exhibits minimal subchronic toxicity in rats.

Notably, our study differs in three critical aspects that enhance its scientific and regulatory relevance: First, the D‐allulose tested herein was derived from a novel one‐step fermentation process using GM *E. coli* AS10, whereas previous studies utilized enzyme‐catalyzed or non‐GM microbial fermentation products [38, 39]. This distinction addresses the unmet need for safety data on process‐specific impurities (e.g., recombinant protein residues and nucleic acid fragments) inherent to GM‐based fermentation, confirming that such impurities do not alter the core toxicological profile of D‐allulose. Second, our highest dose (8000 mg/kg BW/day) exceeded the maximum dose of Matsuo et al. [38] (5000 mg/kg BW/day) and was substantially higher than that of Chen et al. [39] (1670 mg/kg BW/day), leading to a higher established NOAEL (8000 mg/kg BW/day vs. 5000 mg/kg BW/day) and providing more robust evidence for the safety margin of D‐allulose. Third, we employed feed incorporation as the exposure route (mimicking real‐world dietary intake) instead of gavage (commonly used in An et al.), generating ecologically valid data that better reflects human consumption scenarios.

In summary, our results reinforce the consistency of D‐allulose’s safety across different production processes and exposure paradigms. The higher dose range, novel GM‐based fermentation context, and diet‐mimetic exposure route provide complementary data that strengthen the comprehensiveness of D‐allulose’s toxicological evaluation, thereby offering valuable support for its regulatory approval and safe application in food products.

## 5. Conclusion

This study was conducted to address the critical need for the subchronic toxicological evaluation of D‐allulose produced by a novel one‐step fermentation process using GM *E. coli* AS10, a promising production approach for this low‐calorie sweetener with growing food industry potential. Through a 90‐day dietary toxicity study in rats, we systematically assessed a comprehensive panel of toxicological metrics, including general clinical status, growth performance, hematology, serum biochemistry, organ weights, and histopathology, to determine the safety profile of the test substance and the biological significance of any observed parametric changes. Core findings from this study confirm the absence of any treatment‐related mortality, overt clinical signs of toxicity, or histopathological lesions in rats across all D‐allulose dose groups, even at the highest exposure level of 8000 mg/kg BW/day.

Beyond confirming the general low subchronic toxicological profile of D‐allulose, this study delivers three uniquely valuable contributions to its safety assessment and regulatory validation: First, it fills the data gap for process‐specific safety of D‐allulose from GM microbial fermentation, verifying that impurities inherent to this novel process do not alter the core safety of the final product; second, the higher maximum exposure dose provides more robust evidence for an expanded safety margin of D‐allulose, strengthening confidence in its safe use at relevant human dietary levels; third, the dietary exposure paradigm generates ecologically valid data that is more applicable to human consumption scenarios than the gavage route used in previous studies. Practically, these findings not only validate the safety of D‐allulose derived from this innovative GM fermentation process, providing critical experimental evidence to support its regulatory evaluation and potential authorization for wide application in food and beverage products, but also reaffirm the weight‐modulating pharmacological activity of D‐allulose, a desirable attribute for its targeted use in obesity management and metabolic health‐focused food formulations. Collectively, the results of this study solidify the scientific basis for the safe development and application of GM‐fermented D‐allulose, while the high NOAEL and diet‐mimetic exposure data further enhance the comprehensiveness of the toxicological evidence base for D‐allulose as a novel food ingredient.

## Author Contributions

Zinan Li: conceptualization, methodology, validation, formal analysis, investigation, data curation, writing–original draft, writing–review and editing, and project administration. Yanmin Nie: investigation and writing–review and editing. Hongju Du: investigation. Wenjing Zhang: investigation. Shan Zheng: investigation. Wenshu Cong: investigation. Ying Feng: resources. Guojun Li: conceptualization. Haiming Jing: conceptualization and methodology. Junyu Ning: conceptualization, methodology, formal analysis, writing–original draft, writing–review and editing, supervision, and project administration. Shan Gao: conceptualization, methodology, validation, writing–review and editing, supervision, project administration, and funding acquisition.

## Funding

This study was funded by the Beijing Science and Technology Planning Project, 10.13039/501100012401, Z231100004523001.

## Conflicts of Interest

The authors declare no conflicts of interest.

## Supporting Information

Additional supporting information can be found online in the Supporting Information section.

## Supporting information


**Supporting Information 1** Supporting Information: Supporting Information and Methods.

**Supporting Information 2** Supporting Information: Supporting Results and Table.

**Supporting Information 3** Supporting Information: Supporting Figures.

**Supporting Information 4** Supporting Information: Checklist according to the ARRIVE Guidelines.

## Data Availability

The data that support the findings of this study are available on request from the corresponding author. The data are not publicly available due to privacy or ethical restrictions.
