# Supplementary material for: Toxicological Assessment of D‐Allulose From a Novel One‐Step Fermentation Process Using Genetically Modified Escherichia coli: A 90‐Day Dietary Toxicity Study in Rats
Source: J Toxicol. 2026 Mar 3;2026:6597561. doi: 10.1155/jt/6597561 (PMC12957887; doi:10.1155/jt/6597561)
Supplement: Supplementary file 1 — Supporting Information 1 Supporting Information: Supporting Information and Methods. [file JT-2026-6597561-s004.docx]

**Supplemental Materials and Methods**

This document provides detailed experimental information for the detection methods described in the main manuscript’s *Material and Methods* section, including specific instruments, matched reagents, and standardized operating procedures. All reagents used are clinically registered in vitro diagnostic (IVD) products, complying with national and international quality standards (e.g., ISO 13485). All operations were performed in accordance with the manufacturers’ instructions and Good Laboratory Practice (GLP) guidelines to ensure the reliability and reproducibility of results.

**1. Urinary Biochemical Detection**

**1.1 Detection Purpose**

Quantitative analysis of key urinary biochemical indicators for evaluating renal function, metabolic status, and urinary tract integrity.

**1.2 Instrument**

- Model: Bayer CLINITEK-500 urinalysis analyzer
- Manufacturer: Bayer Healthcare LLC, USA

**1.3 Reagents**

- Product Name: SIEMENS Multistix 10 SG Reagent Strips
- Manufacturer: Siemens Healthcare Diagnostics Inc., USA
- Specification: 10-parameter detection (glucose, bilirubin, ketone bodies, specific gravity, blood, pH, protein, urobilinogen, nitrite, leukocytes)

**1.4 Operating Procedure**

Urine samples were collected using metabolic cages. The reagent strip was immersed in the supernatant for 1 s, excess liquid was blotted with filter paper, and the strip was inserted into the analyzer. Detection results were automatically recorded after 60 s, following the instrument’s built-in calibration protocol.

**2. Hematological Detection**

**2.1 Detection Purpose**

Analysis of peripheral blood cell composition (leukocytes, erythrocytes, platelets, etc.) and related parameters to evaluate systemic hematopoietic function and inflammatory responses.

**2.2 Instrument**

- Model: Sysmex XT-2000i automated hematology analyzer
- Manufacturer: Sysmex Corporation, Japan

**2.3 Reagents and Calibration Materials**

| Reagent Name | Manufacturer | Application |
| --- | --- | --- |
| CELLPACK Diluent | Sysmex Corporation, Japan | Sample dilution |
| CELLCLEAN Cleaning Solution | Sysmex Corporation, Japan | Instrument pipeline cleaning |
| SULFOLYSER SLS Hemoglobin Reagent | Sysmex Corporation, Japan | Hemoglobin detection |
| STROMATOLYSER-4DL Leukocyte Classification Lysing Reagent | Sysmex Corporation, Japan | Leukocyte classification (granulocytes/lymphocytes) |
| STROMATOLYSER-4DS Stain for Leukocyte Classification and Reticulocytes | Sysmex Corporation, Japan | Leukocyte staining and reticulocyte detection |
| STROMATOLYSER-FB Basophil Lysing Reagent | Sysmex Corporation, Japan | Basophil separation and counting |
| RETSEARCH (Ⅱ) Reticulocyte Reagent | Sysmex Corporation, Japan | Reticulocyte labeling and quantification |
| SCS-1000 Calibrator | Sysmex Corporation, Japan | Instrument calibration |
| E-CHECKL2 Control Material | Sysmex Corporation, Japan | Quality control of detection results |

**2.4 Operating Procedure**

Whole blood samples were collected in EDTA-K2 anticoagulant tubes and gently inverted 5 times to ensure uniform mixing. Within 2 h of collection, detection was initiated following the “Complete Blood Count + Reticulocyte” protocol. Calibration was performed using SCS-1000 Calibrator before sample detection, and quality control was verified with E-CHECKL2 Control Material.

**3. Coagulation Detection**

**3.1 Detection Purpose**

Measurement of coagulation function indicators (prothrombin time, activated partial thromboplastin time, fibrinogen, etc.) to assess the intrinsic and extrinsic coagulation pathways.

**3.2 Instrument**

- Model: Sysmex CA-1500 automated coagulation analyzer
- Manufacturer: Sysmex Corporation, Japan

**3.3 Reagents, Consumables, and Controls**

| Item Name | Manufacturer | Specification | Application |
| --- | --- | --- | --- |
| Reaction Cups for Automated Coagulation Analyzer | Siemens Healthineers, Germany | SU40 | Sample reaction vessel |
| Calibrator | Siemens Healthineers, Germany | Lyophilized | Instrument calibration |
| Dade Owren's Veronal Buffer | Siemens Healthineers, Germany | 500 mL/bottle | Reaction buffer |
| Dade Actin Activated Cephaloplastin Reagent | Siemens Healthineers, Germany | Lyophilized | Activated partial thromboplastin time (APTT) detection |
| Calcium Chloride Solution | Siemens Healthineers, Germany | 0.025 mol/L | Coagulation initiation reagent |
| Test Thrombin Reagent | Siemens Healthineers, Germany | Lyophilized | Thrombin time (TT) detection |
| Thromborel S | Siemens Healthineers, Germany | Lyophilized | Prothrombin time (PT) detection |
| Dade Ci-Trol 1 Control Material | Siemens Healthineers, Germany | Lyophilized | Quality control (normal range) |
| Dade Thrombin Reagent | Siemens Healthineers, Germany | Lyophilized | Fibrinogen (FIB) quantitative detection |

**3.4 Operating Procedure**

Citrated whole blood samples (9:1 blood-to-anticoagulant ratio) were centrifuged at 3000 rpm for 15 min to obtain platelet-poor plasma. 50 μL of plasma was added to the reaction cup, preheated at 37℃ for 3 min, and corresponding reagents were added according to the detection index. Coagulation time was automatically recorded by the analyzer, with calibration performed using the provided calibrator before detection.

**4. Serum Biochemical Detection**

**4.1 Detection Purpose**

Quantification of serum liver function, renal function, electrolytes, and metabolic indicators to evaluate organ function and systemic metabolic status.

**4.2 Instrument**

- Model: Hitachi 7600 automated biochemistry analyzer
- Manufacturer: Hitachi High-Technologies Corporation, Japan

**4.3 Reagent Kits**

| Reagent Kit Name | Manufacturer | Detection Indicators |
| --- | --- | --- |
| Standard Liver Function 8-Panel Reagent Kit | Tellgen Corporation, China | Alanine aminotransferase (ALT), aspartate aminotransferase (AST), total bilirubin (TBIL), direct bilirubin (DBIL), alkaline phosphatase (ALP), gamma-glutamyl transferase (GGT), total protein (TP), albumin (ALB) |
| Basic Renal Function 3-Panel Reagent Kit | Zhong Sheng Bei Kong Biotechnology Co., Ltd., China | Urea nitrogen (Urea), creatinine (Cr), uric acid (UA) |
| Comprehensive Metabolism 6-Panel Reagent Kit | Tellgen Corporation, China | Total cholesterol (TC), triglycerides (TG), high-density lipoprotein cholesterol (HDL-C), low-density lipoprotein cholesterol (LDL-C), glucose (GLU), lactate dehydrogenase (LDH) |
| Basic Electrolyte 3-Panel Reagent Kit | Kehua Bio-Engineering Co., Ltd., China | Sodium (Na⁺), potassium (K⁺), chloride (Cl⁻) |

**4.4 Operating Procedure**

Serum samples were obtained by centrifuging whole blood at 3000 rpm for 10 min. The analyzer was calibrated using manufacturer-provided calibrators, and quality control was performed with commercial control materials (normal and abnormal levels). The detection was performed following the kit instructions, with reaction temperatures maintained at 37℃.
