# Supplementary material for: Toxicological Assessment of D‐Allulose From a Novel One‐Step Fermentation Process Using Genetically Modified Escherichia coli: A 90‐Day Dietary Toxicity Study in Rats
Source: J Toxicol. 2026 Mar 3;2026:6597561. doi: 10.1155/jt/6597561 (PMC12957887; doi:10.1155/jt/6597561)
Supplement: Supplementary file 2 — Supporting Information 2 Supporting Information: Supporting Results and Table. [file JT-2026-6597561-s003.docx]

**Supplemental Materials for “Toxicological Assessment of D-Allulose from a Novel One-Step Fermentation Process Using Genetically Modified *Escherichia coli*: A 90-Day Oral Feeding Study in Rats”**

**Supplementary Results S1: Detailed serum biochemical analysis**

In the serum biochemical analysis, the toxicological implications of each statistically significant parameter were discussed and evaluated individually as follows. The historical reference ranges for the biochemical parameters are provided in Supplementary Table S1.

1. AST: A moderate increase (40.9%) in AST levels was noted in high-dose male animals as compared to the negative control, which was statistically significant (P<0.05). However, this change did not exceed the historical reference range of our laboratory (92.7~268.3 U/L), and no corresponding histopathological changes were observed in the liver, therefore was not considered toxicologically relevant.
2. TP: A slight increase (3.4%) in TP levels was observed in low-dose male animals, which was statistically significant (P<0.05). Given the small magnitude of change, its occurrence in only one sex and dose group, and lack of a distinct dose-response relationship, this change was not considered toxicologically significant.
3. Alb: A minor increase (7.1%) in Alb levels was noted in high-dose male animals, which was statistically significant (P<0.05). The change was minor, occurred in only one gender and dose group, and did not exhibit a significant dose-response relationship or exceed the historical reference range (32.67~37.07 g/L), thus not deemed toxicologically relevant.
4. Glu: A mild decrease in Glu levels was observed in female animals of low- (15.2%) and mid- (26.4%) dose group, which was statistically significant (P<0.05). The physiological significance of this decrease was considered minimal, as such was not attributed to the toxic effects of D-allulose.
5. TG: An increase (85.2%) in TG levels was noted in female animals of low-dose group, which was statistically significant (P<0.05). This change was observed only in one sex and dose-group, did not show a significant dose-response relationship, and remained within the historical reference range (0.24~2.02 mmol/L), thus was not considered toxicologically significant.
6. TC, LDL-C: Dose-dependent increases (29.6%~81.6%) in TC and LDL-C levels were observed in male animals across low-, mid-, and high-dose groups, which were statistically significant (P<0.05). The changes in TC were within the historical reference range (1.55~2.41 mmol/L), thus not considered a toxic effect of D-allulose.
7. Cr: A mild decrease in Cr levels was observed in female animals of mid- (15.6%) and high -dose group (16.9%), which was statistically significant (P<0.05). The physiological significance of this decrease was considered minimal, thus not attributed to the toxic effects of D-allulose.
8. Urea: Male animals in the high-dose group showed a mild increase (20.1%) in urea levels, which was statistically significant (P<0.05). This change was observed only in one gender and dose group, did not show a clear dose-response relationship, and remained within the historical reference range (5.64~8.11 mmol/L), with no corresponding histopathological changes observed in the kidney, thus not considered toxicologically significant.
9. K^+^, Na^+^: A slight decrease in K^+^ concentration was observed in male animals of mid- (9.6%) and high-dose group (7.7%), respectively. Male animals of high-dose (1.9%) and female animals of low- dose (1.3%) showed a slight decrease in Na+ concentration, both of which were statistically significant (P<0.05). However, these changes are within the historical reference ranges (K^+^ female: 3.82~5.04 mmol/L, K^+^ male: 4.41~5.96 mmol/L, Na^+^ female: 145.43~152.34 mmol/L, Na^+^ male: 146.57~168.96 mmol/L), thus are not considered toxicologically significant.
10. ALP: Female animals in the high-dose group and male animals in both the mid- and high-dose groups showed a statistically significant (P<0.05) rise in ALP levels (42.5%~122.1%). The change in this parameter in mid- and high-dose male animals exceeded the historical reference range (44.04~101.66 U/L) and demonstrated a certain dose-response relationship. However, this elevation in ALP levels lacked toxicological significance, as no corresponding histopathological changes were observed in the kidneys, liver, bones, or intestines.

**Supplementary Table S1 Historical reference value range of** **biochemistry assessment parameters (95% confidence interval)**

| **Parameters** | **Female** | **Male** |
| --- | --- | --- |
| **ALT (U/L)** | 21.4~59.6 | 26.01~68.24 |
| **AST (U/L)** | 81.2~201.8 | 92.72~268.28 |
| **ALP (U/L)** | 23.6~61.4 | 44.0~101.6 |
| **TP (g/L)** | 59.7~69.5 | 58.82~67.42 |
| **Alb (g/L)** | 34.4~40.6 | 32.67~37.07 |
| **Glu (mmol/L)** | 4.20~7.18 | 4.31~7.42 |
| **TG (mmol/L)** | 0.24~2.02 | 0.38~2.24 |
| **TC (mmol/L)** | 1.17~1.95 | 1.55~2.41 |
| **Cr (umol/L)** | 37.93~53.16 | 40.18~58.88 |
| **Urea (mmol/L)** | 4.98~8.09 | 5.64~8.11 |
| **K^+^ (mmol/L)** | 3.82~5.04 | 4.41~5.96 |
| **Na^+^ (mmol/L)** | 145.43~152.34 | 146.57~168.96 |
| **Cl^+^ (mmol/L)** | 103.77~109.55 | 100.91~115.8 |

The data were obtained from 102 rats of each gender in the negative control groups of 90-day repeated oral toxicity tests conducted in our laboratory over the past decade.

**Supplementary Results S2: Detailed histopathological examination**

For each organ or tissue, detailed descriptions of pathological observations are provided as follows.

1. Brain, Cerebellum: In both the negative control and high-dose groups, the brain and spinal cord tissues of male and female animals exhibited clear structural delineation in all layers and regions, with no abnormalities in cell morphology, number, or ratio. There were no signs of degeneration, necrosis, hemorrhage, inflammation, or aberrant proliferation.
2. Spinal Cord: In the negative control and high-dose groups, the gray and white matter of the spinal cord in male and female animals were clearly demarcated, with no evidence of neuronal degeneration or necrosis.
3. Pituitary Gland: In the negative control and high-dose groups, the adenohypophysis and neurohypophysis of male and female animals were clearly demarcated, with no abnormalities in composition, morphology or ratio of adenocytes, and no evidence of adenocyte hyperplasia.
4. Heart: In one male animal (1/12) from the negative control group, irregularly shaped foci of chronic inflammatory cell infiltration, predominantly lymphocytes and monocytes, were observed in the interstitial myocardium of the left ventricular free wall and the subendocardium of the right ventricular free wall. Similar inflammatory cell infiltration was observed in the interstitial myocardium near the epicardium of the right ventricular free wall in one male animal (1/12) from the high-dose group. These were considered as spontaneous lesions. There was no significant degeneration, necrosis, atrophy, or hypertrophy of myocardial fibers in other male and female animals from both the high-dose and negative control groups. There was no significant epicardium and endocardial hyperplasia, no inflammatory cell infiltration in the interstitium, and no proliferation of fibrous connective tissue.
5. Liver: In male (10/12) and female (6/12) animals from the negative control group, as well as male (3/12) and female (11/12) animals from the high-dose group, focal infiltration of lymphocytes and monocytes was observed in the liver tissue, which was mild and considered a spontaneous lesion. In male (1/12) and female (7/12) animals from the negative control group, as well as male animals (5/12) from the high-dose group, scattered fatty degeneration of hepatocytes was observed in the liver tissue, which was also considered a spontaneous lesion. No structural abnormalities in liver tissue or hepatocyte differentiation were observed in animals from all groups, with no evidence of hepatocyte proliferative changes and no proliferation of bile ducts or connective tissue in the portal areas.
6. Spleen: In both high-dose and control groups, spleens of animals had intact capsules, with normal structures of the splenic trabeculae, red pulp, white pulp, and marginal zones, appropriate proportions, and no atrophy or abnormal hyperplasia of lymphoid nodules.
7. Lungs: In one male animal (1/12) from the negative control group, a large number of foam cells were observed in focal alveoli; and in another male animal (1/12) from the negative control group, focal calcification was observed in the lung tissue near the pleura. Both were considered spontaneous lesions. In other male and female animals from the negative control and high-dose groups, the alveolar cavities and bronchial cavities at all levels were clear, with no significant exudation or hemorrhage. No degeneration, necrosis, hyperplasia, or metaplasia of tracheal and bronchial epithelial cells, alveolar epithelial cells, and no fibrous changes in the interstitium were observed.
8. Kidneys: All animals in the high-dose and negative control groups had intact renal capsules with clear boundaries between the cortex and medulla. The glomeruli had normal structure and were evenly distributed in the cortical area. The morphology and number of various types of cells in the glomerular cross-section were normal. There were no casts in the renal tubular lumen, no significant epithelial degeneration or necrosis, and no proliferation of connective tissue or inflammatory cell infiltration in the interstitium.
9. Adrenal Glands: In both negative control and high-dose groups, the adrenal cortex and medulla of male and female animals had clear boundaries between the layers of tissue structure, with appropriate cell proportions, and no lesions such as hemorrhage, necrosis, atrophy, or hyperplasia were observed.
10. Thyroid Gland: In one male animal (1/12) from the high-dose group, focal lymphocytic infiltration of the thyroid gland was observed, which was considered a spontaneous lesion. Ectopic thymic tissue, an aberration of embryonic development, was seen locally in the thyroid gland of another male animal (1/12) from the high-dose group. In other male and female animals from both the negative control and high-dose groups, no inflammation or fibrosis was observed in the thyroid interstitium. The morphology, size, and structure of the thyroid follicles were normal, the colloid content was appropriate, and no lesions such as hemorrhage, necrosis, atrophy, or abnormal hyperplasia were observed.
11. Thymus: In three female animals (3/12) from the negative control group, one male animal (1/12) from the high-dose group, and six female animals (6/12) from the high-dose group, focal epithelioid cell proliferation was observed locally in the thymus, which was considered a spontaneous lesion. The remaining male and female animals in the negative control group and the high-dose group had clear structures of the thymic cortex and medulla, with no abnormalities in the morphology or number of thymocytes.
12. Lymph Nodes: In male and female animals from both the negative control and high-dose groups, the cortex and medulla of the lymph nodes had clear boundaries and appropriate proportions. The structure of the lymphoid nodules was clear, with no atrophy, abnormal hyperplasia, or necrosis observed.
13. Pancreas: Focal vacuolar changes in the epithelium of the acinar exocrine part of the pancreas were observed in one male animal (1/12) and three female animals (3/12) from the negative control group, as well as one female animal (1/12) from the high-dose group. In one female animal (1/12) from the negative control group and one male animal (1/12) from the high-dose group, focal atrophy of the acinar exocrine part of the pancreas was observed. Both were considered spontaneous lesions. The remaining male and female animals in both the negative control and high-dose groups had clear structures of the acinar, ductal, and islet parts of the exocrine pancreas, with no hemorrhage, necrosis, inflammation, fibrosis, or abnormal hyperplasia observed.
14. Esophagus: In all animals of both negative control and high-dose group, the mucosal layer, submucosal layer, muscular layer, and adventitial layer of esophagus had normal structures. The stratified epithelium of the mucosal layer showed no abnormal differentiation, no hemorrhage, erosion, or ulceration, and no abnormal hyperplasia.
15. Gastrointestinal Tract: Histopathological examination of the gastrointestinal mucosa, submucosa, muscularis, and serosa layers in male and female animals of both the negative control and high-dose groups showed normal histological architecture. The mucosal epithelium exhibited no signs of aberrant differentiation, and there was an absence of pathological conditions such as hemorrhage, erosion, ulceration, or neoplastic proliferation. The lamina propria and muscularis mucosae did not demonstrate any dysplastic changes, indicating the preservation of gastrointestinal integrity and function.
16. Bladder: In both the negative control and high-dose groups, male and female animals exhibited distinct stratification of the bladder mucosal epithelium, lamina propria, and muscularis layers. The urothelial cells lacked any signs of aberrant differentiation or hyperplasia, and there was an absence of inflammatory pathologies.
17. Salivary Glands: In both the negative control and high-dose groups, male and female animals demonstrated normal acinar and ductal architecture within the salivary glands. No evidence of hemorrhage, necrosis, inflammation, fibrosis, or dysplastic changes was observed.
18. Eyeballs: Male and female animals in both the negative control and high-dose groups showed well-defined layers of the retina with no alterations in the proportional composition. The fundus oculi was devoid of any hemorrhagic or angiogenic abnormalities.
19. Skeletal Muscle: Male and female animals in both the negative control and high-dose groups showed clear fascicular patterns with distinct myofibrillar striations. The nuclei were normally distributed and dense, with no indications of muscle atrophy, degeneration, or inflammatory infiltrates.
20. Sciatic Nerve: In both the negative control and high-dose groups, male and female animals displayed no hemorrhagic or inflammatory changes in the epineurium or nerve fiber bundles. The nerve fibers maintained their structural integrity, with no evidence of axonal degeneration or demyelination.
21. Femur, Joints, and Bone Marrow: Male and female animals in both the negative control and high-dose groups exhibited clear delineation of cortical and trabecular bone structures, as well as articular cartilage, with appropriate lamellar bone width. There was no evidence of abnormal ossification or osteoclastic activity. The chondrocytes of the articular surfaces were uniform in morphology and density, with no significant chondrocyte degeneration, necrosis, or hyperplasia, and the cartilage matrix was uniformly distributed. The synovium showed no evidence of degeneration, necrosis, inflammation, or angiogenic changes. The medullary cavity was filled with hematopoietic red marrow, which had appropriate ratios of hematopoietic lineages and a high concentration of blood cells at various stages of maturation.
22. Testes: In one male animal (1/12) from the negative control group, bilateral testicular atrophy was observed, which was considered an incidental finding. The remaining male animals from both the negative control and high-dose groups showed well-developed epithelium in all stages of the seminiferous tubules, with mature spermatozoa present. There was no evidence of hyperplasia in Sertoli cells or interstitial cells of the testes, and the tunica albuginea did not exhibit any thickening.
23. Epididymis: Male animals in both the negative control and high-dose groups had no anatomical abnormalities in their epididymal and efferent ducts. Except for the individual with testicular atrophy, the ductal lumen was full of mature spermatozoa.
24. Prostate: In four male animals (4/12) from the negative control group and one male animal (1/12) from the high-dose group, focal chronic interstitial prostatitis was observed, which was considered an incidental finding. The remaining male animals in the negative control and high-dose groups exhibited no anatomical abnormalities in the prostate gland, with no inflammation or hyperplasia, or abnormal differentiation of epithelial cells.
25. Uterus: In one female animal (1/12) from the negative control group and three female animals (3/12) from the high-dose group, uterine cavity dilation was observed, which was considered an incidental finding. The remaining female animals in both the negative control and high-dose groups exhibited intact uterine layer structures and no endometrial atrophy or hyperplasia.
26. Ovaries: In female animals from both the negative control and high-dose groups, the ovaries contained an appropriate number and proportion of follicles and corpora lutea at various stages of development, with no evidence of haemorrhage, cyst formation, or fibrosis.
