# Supplementary material for: Toxicological Assessment of D‐Allulose From a Novel One‐Step Fermentation Process Using Genetically Modified Escherichia coli: A 90‐Day Dietary Toxicity Study in Rats
Source: J Toxicol. 2026 Mar 3;2026:6597561. doi: 10.1155/jt/6597561 (PMC12957887; doi:10.1155/jt/6597561)
Supplement: Supplementary file 3 — Supporting Information 3 Supporting Information: Supporting Figures. [file JT-2026-6597561-s002.pdf]

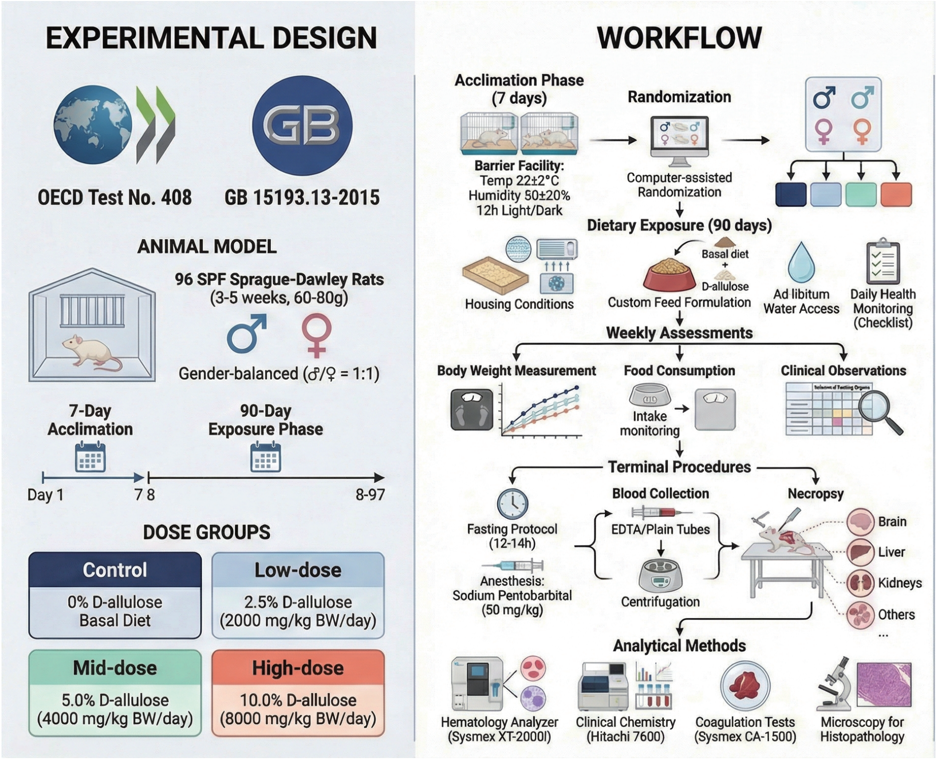

Supplemental Figure 1. Experimental desigh and workflow

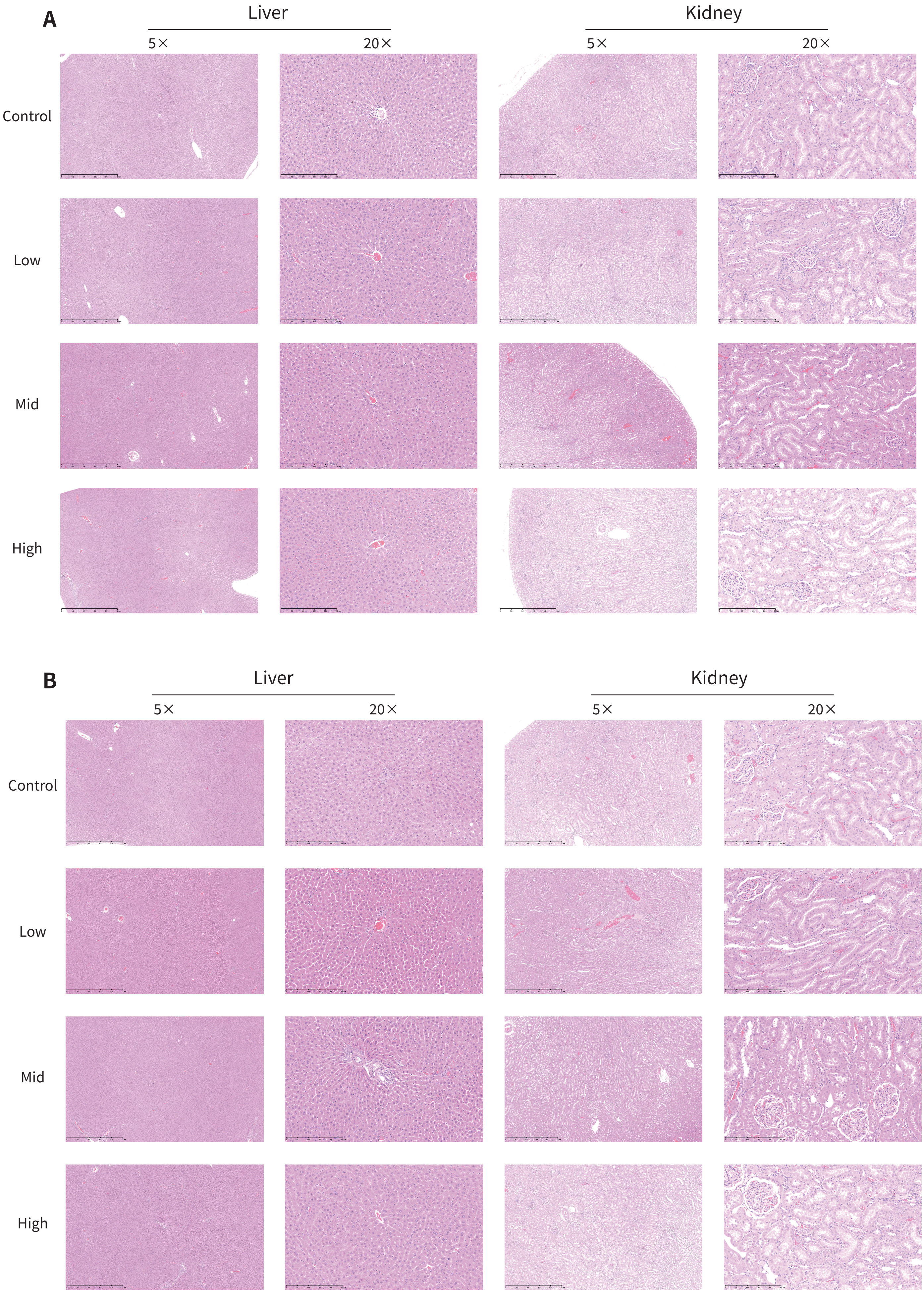

Supplemental Figure 2. Representative H&E-stained sections of liver and kidney tissues from female (A) and male (B) SPF-grade SD rats in the 90-day subchronic oral toxicity study of D-allulose produced by genetically modified Escherichia coli AS10.
